# Gold‐Dithiocarbamato Glycoconjugates as Potential Anticancer Agents: Design, Physico‐Chemical Characterization, and *In Vitro* Biological Activity

**DOI:** 10.1002/cbic.202500447

**Published:** 2025-08-26

**Authors:** Andrea Pettenuzzo, Jessica Wölker, Luciano Marchiò, Ingo Ott, Luca Ronconi

**Affiliations:** ^1^ School of Biological and Chemical Sciences University of Galway University Road Galway H91 TK33 Ireland; ^2^ Institute of Medicinal and Pharmaceutical Chemistry Technische Universität Braunschweig Beethovenstr., 55 38106 Braunschweig Germany; ^3^ Department of Chemistry, Life Sciences and Environmental Sustainability University of Parma Parco Area delle Scienze 11/a 43124 Parma Italy

**Keywords:** glucose transporters, gold(I)‐*N*‐heterocyclic carbene, gold(I)‐phosphine, gold‐dithiocarbamates, gold‐glycoconjugates

## Abstract

To develop new metal‐based glycoconjugates as potential anticancer agents, three gold(III)‐dithiocarbamato glycoconjugates of the type [Au^III^Br_2_(SSC‐Inp‐GlcN)] (**Au3‐5**), their gold(I)‐phosphine counterparts [Au^I^(SSC‐Inp‐GlcN)(PPh_3_)] (**AuP3‐5**), and gold(I)‐carbene analogs [Au^I^(SSC‐Inp‐GlcN)(Et_2_BzImy)] (**AuC3‐5**) (Inp: isonipecotic moiety; GlcN: amino‐glucose scaffold; Et_2_BzImy: 1,3‐diethylbenzimidazol‐2‐ylidene moiety), as well as the corresponding non‐glycosylated counterparts (**Au1‐2**, **AuP1‐2,** and **AuC1‐2**) bearing a terminal ester or amide function, are generated and characterized by means of several analytical techniques (FT‐IR, ^1^H‐/^13^C‐NMR, UV–Vis, X‐ray crystallography). Their stability under physiologically relevant conditions (phosphate‐buffered saline solution) has also been evaluated. Contrary to the gold(III)‐glycoconjugates, the glucose‐functionalized gold(I) derivatives show a significant antiproliferative effect against colorectal adenocarcinoma (HT‐29), metastatic breast adenocarcinoma (MDA‐MB‐231), and breast adenocarcinoma (MCF‐7) cells, with IC_50_ values in the low micromolar range, the gold(I)‐phosphine derivatives turning up to be the best performers. Cell uptake studies show no evident correlation between cell growth inhibition and cellular uptake, and the use of glucose‐free cell culture media and a GLUT1 inhibitor rules out the involvement of glucose transporters in cell internalization, thus suggesting alternative cell death pathways such as acting at extracellular level (especially for the gold(I) derivatives).

## Graphical abstract

Metal‐dithiocarbamato glycoconjugates based on gold(III), gold(I)‐phosphino, and gold(I)‐carbene scaffolds are here investigated as potential anticancer agents. Contrary to expectations, they induce cell death by acting at the extracellular level rather than targeting overexpressed glucose transporters.

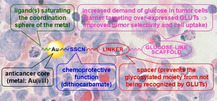

## 1 Introduction

Contrary to ‘normal’ (i.e., healthy) cells, neoplastic cells (either benign or cancerous) are characterized by excessive growth and replication, which make them in constant demand of nutrients and energy to sustain such abnormal proliferation rates.^[^ ^1^ ^]^ As a result, tumor cells undergo major metabolic reprogramming aimed at gaining a competitive advantage over neighboring stromal and immune cells to secure the resources necessary to their survival in an hostile environment.^[^ ^2^ ^]^ Among the various hallmarks of cancer identified to date,^[^ ^3^ ^]^ the so‐called ‘Warburg effect’ is one of the most studied alterations of tumor metabolism. First postulated by Otto Warburg in 1924,^[^ ^4^ ^]^ this phenomenon is based on the observation that the glucose metabolism in cancer cells favors glycolysis over oxidative phosphorylation to generate energy even in presence of adequate supply of oxygen.^[^ ^5^ ^]^ Such process (known as aerobic glycolysis) is indeed less efficient in terms of amount of energy produced (only 2 adenosine triphosphates (ATPs) produced per molecule of glucose), but it has the advantage of generating energy faster, and is a common metabolic feature in many types of cancers.^[^ ^6^ ^]^

A major consequence of such altered glucose metabolism is the over‐expression of glucose transporters (GLUTs), thus allowing cancer cells to outcompete other cells for increasing glucose uptake.^[^ ^7^ ^]^ Moreover, it leads to the overproduction of lactate which has a major impact on the tumor microenvironment (TME), including its increased acidity.^[^ ^8^ ^]^ Consequently, the up‐regulation of GLUTs and the altered TME would represent suitable targets for the development of anticancer therapeutics and diagnostic drugs.^[^ ^9^ ^,^ ^10^ ^]^ In this regard, the rational design of glyco‐functionalized small molecules having tumor‐targeting properties has been shown promise. Carbohydrates necessary to fulfill the body's energy needs are internalized inside the cell through GLUTs, transport proteins facilitating the translocation of sugars (especially glucose) and glyco‐mimetics across cell membrane.^[^ ^11^ ^]^ To date, 14 GLUTs (GLUT1 to GLUT14) have been identified, each showing preferential affinity and recognition of specific monosaccharides.^[^ ^12^ ^]^ Although such transporters are expressed in nearly every type of cells, most tumor cells over‐express several GLUTs (in particular GLUT1)^[^ ^13^ ^]^ to support aerobic glycolysis and the associated increased demand for glucose. Therefore, glycosylation of known anticancer scaffolds turns out to be a promising approach to the generation of tumor‐targeting pro‐drugs in which the monosaccharide pendant would act as a carrier targeting GLUTs over‐expressed in cancer cells. Should the sugar moiety be recognized, the glycoconjugate would be transported inside the cell as a whole and, once internalized, the actual bioactive substance would then be released thus exerting its anticancer activity directly from the inside of the tumor cell.^[^ ^14^ ^]^ Even though, to the best of our knowledge, no such therapeutic targeting system is yet available in the market, this ‘Trojan Horse’ approach has proved experimentally successful upon glycosylating clinically established anticancer drugs, such as Chlorambucil (functionalized with d‐threose), Paclitaxel (with d‐glucose), and Doxorubicin (with d‐galactose or d‐glucose) among others.^[^ ^15^ ^]^

Within this research field, coordination compounds are well‐known to exert varied biological properties (including anticancer activity) and mechanisms of action not achievable by purely organic compounds owing to the wide range of oxidation states, coordination geometries and reactivity of the metal centers, as well as their capability to be bound by a virtually unlimited number and type of ligands. The rational design of metal complexes would allow for the fine‐tuning of their bioactivity, but their full potential has yet to be achieved.^[^ ^16^, ^17^ ^–^ ^18^ ^]^ In this context, metal‐glycoconjugates are largely underrepresented. Although some metal complexes functionalized with carbohydrates (mainly glucose and glucose‐like scaffolds) were investigated as targeted anticancer and diagnostic agents,^[^ ^19^ ^,^ ^20^ ^]^ to the best of our knowledge, very few examples have been reported in recent years, mostly related to platinum,^[^ ^21^ ^,^ ^22^ ^]^ ruthenium,^[^ ^23^, ^24^ ^–^ ^25^ ^]^ and osmium^[^ ^25^ ^]^ derivatives, as well as for antimicrobial applications.^[^ ^26^ ^]^ Moreover, with few exceptions, the majority of studies have been confined to routine cytotoxicity evaluations, while comprehensive investigations into the relationship between anticancer activity, cellular uptake, and the ability to target GLUTs have been rarely investigated. For instance, Lippard *et al.* have provided a robust proof‐of‐concept by demonstrating that three rationally designed platinum(II)‐glycoconjugates exhibit pronounced antiproliferative effects across a panel of human tumor cell lines *via* GLUT‐mediated transport pathways, resulting in preferential intracellular accumulation within cancer cells *in vitro*, thereby providing solid validation of the proposed targeting strategy.^[^ ^27^ ^,^ ^28^ ^]^

Among transition metals, gold has been the focus of considerable interest, leading to the design and biological evaluation of a number of related complexes showing potential as chemotherapeutics.^[^ ^29^, ^30^ ^–^ ^31^ ^]^ In this regard, some gold(III)‐dithiocarbamato derivatives stood out for their remarkable anticancer activity both *in vitro* and *in vivo* ^[^ ^32^ ^,^ ^33^ ^]^ coupled with negligible acute and organ toxicity,^[^ ^34^ ^]^ achieved by inhibiting intracellular targets alternative to the clinically‐established platinum drugs (such as the cellular redox metabolism^[^ ^35^ ^]^ and the ubiquitin‐proteasome pathway^[^ ^36^ ^]^). Nonetheless, tumor selectivity remains a significant challenge despite the encouraging results. In an effort to address this challenge, we have previously reported on some glycosylated organogold(III)‐dithiocarbamato complexes whose antiproliferative activity seems to result from their capability to inhibit topoisomerase I and II, although cell uptake seems to be mediated by facilitated diffusion whereas the involvement of glucose transporters was not undoubtedly proved.^[^ ^37^ ^]^

To further advance our research in the field of metal‐glycoconjugation, we report here on the design, synthesis, chemical characterization, and in vitro biological evaluation of a set of gold(III)‐dithiocarbamato glycoconjugates of the type [Au^III^Br_2_(SSC‐Inp‐GlcN)], their gold(I) counterparts [Au^I^(SSC‐Inp‐GlcN)(PPh_3_)], and [Au^I^(SSC‐Inp‐GlcN)(Et_2_BzImy)] (Inp: isonipecotic moiety; GlcN: amino‐glucose scaffold; Et_2_BzImy: 1,3‐diethylbenzimidazol‐2‐ylidene moiety), and the corresponding non‐glycosylated model gold(III/I) complexes. Results are discussed here and compared with existing data in the literature.

## 2 Results and Discussion

### 2.1 Design and Synthetic Strategy

**Figure 1:**
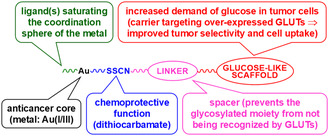
General design of the target gold‐dithiocarbamato glycoconjugates.

All the metal‐glycoconjugates here discussed share the same general structure (**Figure** **1**): a gold‐containing scaffold bearing different ancillary ligands depending on the oxidation state and geometry of the metal center (chemotherapeutic) bound to a glucose‐like scaffold (carrier) through an isonipecotic‐dithiocarbamato linker (spacer) directly coordinated to the metal ion.

**Figure 2:**
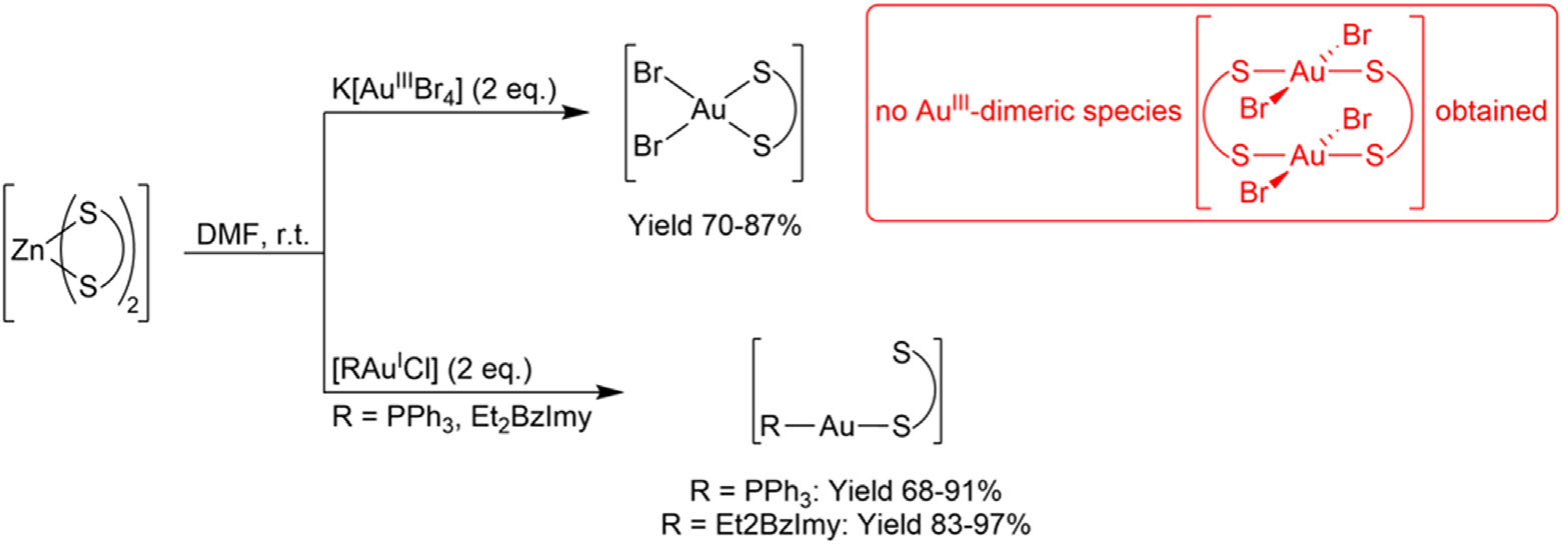
General synthetic route to the generation of the target gold‐dithiocarbamato glycoconjugates *via* transmetallation from the corresponding zinc(II)‐dithiocarbamato intermediates.

Glycoconjugation of (metallo)drugs may be challenging, resulting in multistep reactions, lengthy chromatographic purifications, and low overall yields. To overcome such issues, we have recently developed an efficient synthetic protocol to the glyco‐functionalization of various metal scaffolds which is based on the pregeneration of zinc(II)‐dithiocarbamato glycoconjugate precursors followed by the transmetallation of the dithiocarbamato ligand as a whole to the desired metal center (**Figure** **2**).^[^ ^38^ ^]^ Not only such strategy allowed the straightforward synthesis of the gold‐dithiocarbamato derivatives in high yields and purity, but it was also crucial to avoid the generation of a mixture of both gold(III) monomeric and dimeric species (a common side‐product which is nearly impossible to separate).^[^ ^39^ ^]^

**Figure 3:**
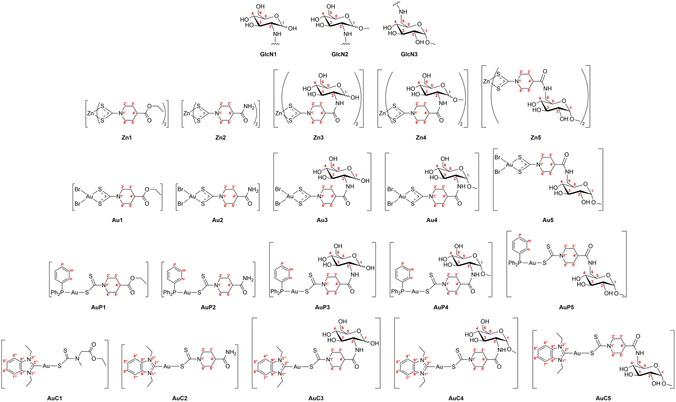
Chemical drawings of the glucose‐like scaffolds, zinc(II)‐dithiocarbamato intermediates, and the corresponding gold(III)‐ and gold(I)‐dithiocarbamato derivatives (with numbering) used in this work.

With reference to **Figure** **3**, three amino‐glucose‐like moieties, namely, 2‐NH‐2‐deoxy‐d‐glucose (**GlcN1**), 1‐*O*‐methyl‐2‐NH‐2‐deoxy‐d‐glucopyranoside (**GlcN2**), and 1‐*O*‐methyl‐6‐NH‐6‐deoxy‐d‐glucopyranoside (**GlcN3**), were chosen for this study, and the corresponding zinc(II)‐dithiocarbamato intermediates **Zn3‐5** were obtained. The {Br_2_Au^III^}‐type complexes **Au3‐5** were synthesized by zinc(II)‐to‐gold(III) transmetallation on account of the promising biological results previously reported for analogous derivatives bearing the same metal‐dithiocarbamato scaffold,^[^ ^32^ ^,^ ^33^ ^]^ with a view to increasing tumor selectivity while retaining their antiproliferative activity toward cancer cells. As to the gold(I) compounds, given the well‐known potential of gold(I)‐phosphine derivatives as anticancer agents,^[^ ^40^ ^]^ the {(Ph_3_P)Au^I^}‐dithiocarbamato analogs **AuP3‐5** were developed. Moreover, since gold(I)‐carbene scaffolds have been gaining great interest as prospective anticancer agents,^[^ ^41^ ^]^ the study was extended to the {(Et_2_BzImy)Au^I^}‐type counterparts **AuC3‐5**. Finally, to evaluate the effect of glycoconjugation on bioactivity, the corresponding model non‐glycosylated metal complexes **Zn1‐2**, **Au1‐2**, **AuP1‐2,** and **AuC1‐2**, respectively, were also generated.

All amino‐sugar precursors (**GlcN1‐3**), zinc(II) intermediates (**Zn1‐5**), gold(III) complexes (**Au1‐5**), and the gold(I)‐phosphine complexes **AuP4‐5** were synthesized as previously reported, and their spectroscopic characterization was fully consistent with literature data (see the Supporting Information for detailed synthetic procedures and assignments).^[^ ^38^ ^]^

### 2.2 Chemical Characterization of the Gold(I)‐Phosphine Complexes

To complete the full set of gold(I)‐phosphine complexes, the model non‐glycosylated derivatives [Au^I^(SSC‐Inp‐OEt)(PPh_3_)] (**AuP1**) and [Au^I^(SSC‐Inp‐NH_2_)(PPh_3_)] (**AuP2**) (featuring either an ester or an amide terminal group, respectively), as well as the corresponding glycoconjugate [Au^I^(SSC‐Inp‐GlcN1)(PPh_3_)] (**AuP3**) functionalized with the commercially available glucosamine scaffold (as a mixture of *α* and *β* anomers), were prepared according to a modified literature procedure.^[^ ^42^ ^]^ The newly synthesized gold(I) complexes were characterized by several analytical and spectroscopic techniques (see the Experimental Section for detailed synthetic procedure and assignments).

A number of bands were consistently recorded for all complexes in the mid‐ and far‐IR spectra, resulting from the common {Ph_3_P‐Au^I^‐(SSC‐Inp)} scaffold, such as the vibrations arising from the coordinated triphenylphosphine ligand at ~1101 (*ν*_*q*__vib_, P—Ph_3_), ~710/69 (*ν*_*r*__vib_, P—Ph_3_), ~540/500 (*δ*_*y*__vib_, P—Ph_3_), ~450/430 (*ν*_*t*__vib_, P—Ph_3_), ~398 (*ν*, Au—P), and ~280/250 (*δ*_*x*__vib_, P—Ph_3_) cm^−1^,^[^ ^43^ ^,^ ^44^ ^]^ as well as the characteristic bands at ~1480 and ~355 cm^−1^ assignable to the N‐CSS and Au‐S stretching modes, respectively.^[^ ^38^ ^]^ As reported in the literature for other triphenylphosphine‐gold(I)‐dithiocarbamato complexes,^[^ ^45^ ^]^ the dithiocarbamato moiety is expected to act as a monodentate ligand binding the gold(I) center through one sulfur atom. This is in agreement with the presence of two bands at *ca.* 1005 and 960 cm^−1^, which are indicative of a monodentate Au—SC(=S)N bonding.^[^ ^46^ ^]^

The other main vibrations are consistent with the different terminal moieties present, that is, the ethyl ester of **AuP1** (*ν*(C=O) = 1729, *ν*(C—OEt) = 1176, *ν*(O—Et) = 1040 cm^−1^), the primary amide group of **AuP2** (*ν*_a,s_(NH_2_) = 3410/3163, *ν*(C=O/amide I) = 1676, *δ*_ip_(CNH_2_/amide II) = 1614 cm^−1^), and the glucose scaffold of **AuP3** bound to the isonipecotic linker through a secondary amide function at the C^2^ position (*ν*(OH + NH overlapped) = 3401, *ν*(C=O/amide I) = 1650, *δ*_ip_(CNH/amide II) = 1544, *ν*(C—OH) = 1060 cm^−1^).^[^ ^38^ ^]^

Mono‐ and multinuclear NMR spectroscopy allowed full characterization of the complexes. The ^1^H, ^13^C, and ^31^P signals associated with the {Ph_3_P‐Au^I^‐(SSC‐Inp)} scaffold of **AuP1‐3** were all recorded at very similar chemical shifts and in agreement with literature data,^[^ ^38^ ^]^ the most diagnostic peak originating from the dithiocarbamato carbon atom at 204‐207 ppm (again indicative of the monodentate coordination of a dithiocarbamato ligand to a gold(I) center).^[^ ^47^ ^]^ As previously discussed in relation to the IR bands, specific peaks accounting for the different terminal moieties were recorded for the ester group of **AuP1** and the primary amide of **AuP2** (e.g., *δ*(***C*** =O) = 174.3 and 176.3 ppm, respectively). As far as complex **AuP3** is concerned, the interpretation of the NMR signals of the glucose‐like fragment proved challenging since nearly all peaks were duplicated and overlapped due to the expected presence of both anomeric species (solution *α*:*β* anomers ratio ≈3.3:1 based on the ^1^H NMR spectrum).

### 2.3 Chemical Characterization of the Gold(I)‐Carbene Complexes

The model non‐glycosylated gold(I)‐carbene derivative [Au^I^(SSC‐Inp‐NH_2_)(Et_2_BzImy)] (**AuC2**) and the corresponding metal‐glycoconjugates [Au^I^(SSC‐Inp‐GlcN1)(Et_2_BzImy)] (**AuC3**), [Au^I^(SSC‐Inp‐GlcN2)(Et_2_BzImy)] (**AuC4**), and [Au^I^(SSC‐Inp‐GlcN3)(Et_2_BzImy)] (**AuC5**) were prepared according to a modified literature procedure (see the Experimental Section for detailed synthetic procedure).^[^ ^42^ ^]^ Contrary to the gold(I)‐phosphine counterpart, the generation of the non‐glycosylated complex featuring a terminal isonipecotic ester proved unsuccessful. Therefore, the alternative complex [Au^I^(SSC‐Sar‐OEt)(Et_2_BzImy)] (Sar: *N*‐methylglycine (sarcosine) moiety; **AuC1**) was obtained, in which the ethyl ester of the sarcosinedithiocarbamato ligand is bound to the gold(I) center (see the Supporting Information and the Experimental Section for detailed synthetic procedures). All gold(I)‐carbene derivatives were characterized by several analytical and spectroscopic techniques (see the Experimental Section for detailed assignments).

**Figure 4:**
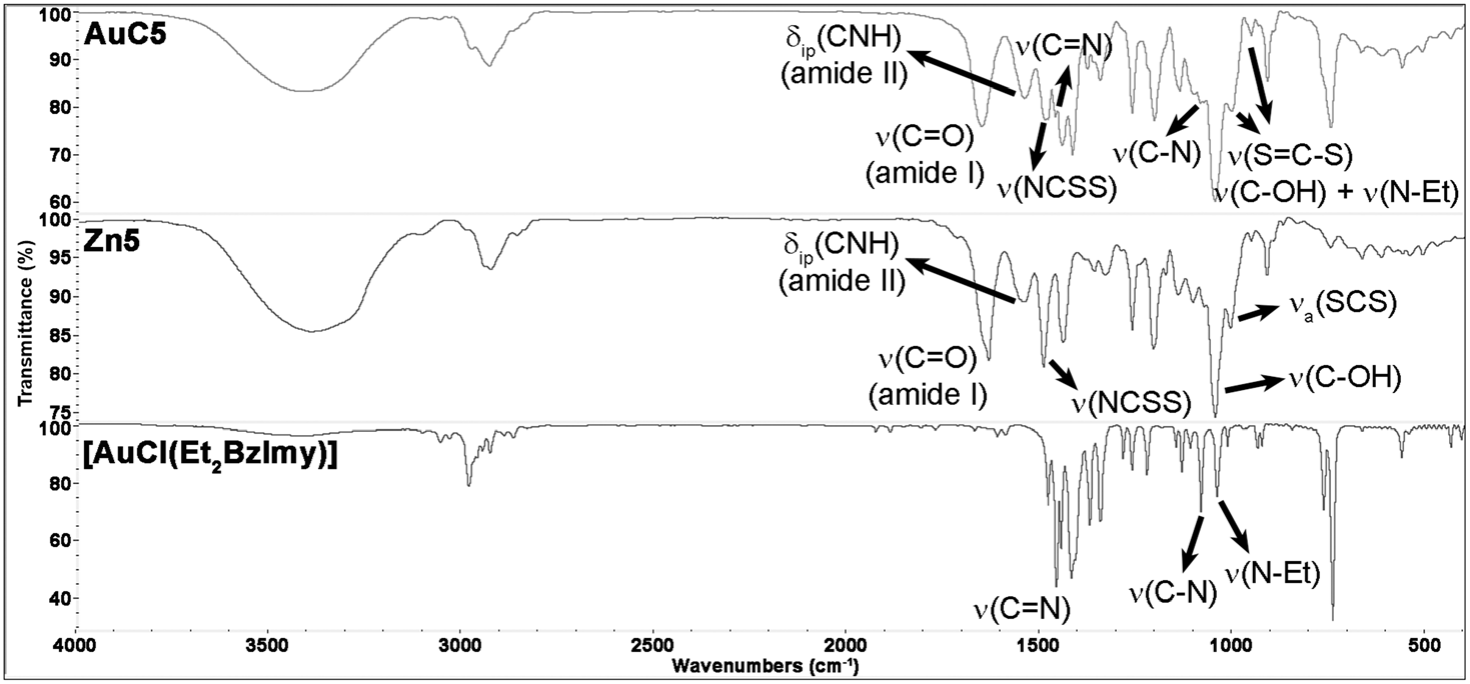
Comparison of the mid‐IR spectra in CsI of [Au^I^Cl(Et_2_BzImy)], [Zn^II^(SSC‐Inp‐GlcN3)_2_] (**Zn5**), and [Au^I^(SSC‐Inp‐GlcN3)(Et_2_BzImy)] (**AuC5**).

Regardless of the terminal pendants, all gold(I) complexes shared similar spectroscopic features resulting from the presence of the same {carbene‐Au^I^‐dithiocarbamato} scaffold. IR spectroscopy provided insights into the identification of the synthesized complexes and the assessment of the coordination mode of the ligands onto the metal center. As an example, the mid‐IR spectrum of the gold(I) complex **AuC5** is shown in **Figure** **4** and compared with those of the gold(I)‐carbene precursor [Au^I^Cl(Et_2_BzImy)] and the corresponding zinc(II) intermediate **Zn5**.

Most spectral features of [Au^I^Cl(Et_2_BzImy)] are generally retained, including *ν*(C=N), *ν*(C—N),^[^ ^48^ ^,^ ^49^ ^]^ and *ν*(N—Et)^[^ ^50^ ^]^ of the carbene ligand at *ca.* 1460, 1085, and 1045 cm^−1^, respectively. Interestingly, the strong characteristic ‘thioureide’ band attributed to the N—CSS stretching vibration is recorded at values (1477‐1492 cm^−1^) similar to those of the zinc(II) precursors (1487‐1494 cm^−1^) but at energies substantially lower than the gold(III) counterparts (1571‐1555 cm^−1^). This is consistent with a reduction of the electron‐withdrawing effect when moving from gold(III) to the less positively charged gold(I) center.^[^ ^38^ ^,^ ^47^ ^]^ In contrast, the force constant of the N‐CSS bond appears to be negligibly affected by the change of charge (from + 2 to + 1) and coordination environment (from bis‐chelate to monodentate dithiocarbamato ligands) upon zinc(II)‐gold(I) transmetallation. Moreover, as previously discussed for the gold(I)‐phosphine derivatives, the presence of two bands at *ca.* 1005 and 960 cm^−1^ in all complexes **AuC1‐5** (assigned to the stretching vibrations of the S=C—S moiety) supports the monodentate coordination of the dithiocarbamato scaffold to the gold(I) center.^[^ ^46^ ^]^ This is in contrast with the single band recorded for all zinc(II) precursors and gold(III) analogs at around 1010 cm^−1^ assigned to the antisymmetric stretching vibration of the SCS group expected for a chelating coordination of dithiocarbamato ligands.^[^ ^51^ ^]^

**Figure 5:**
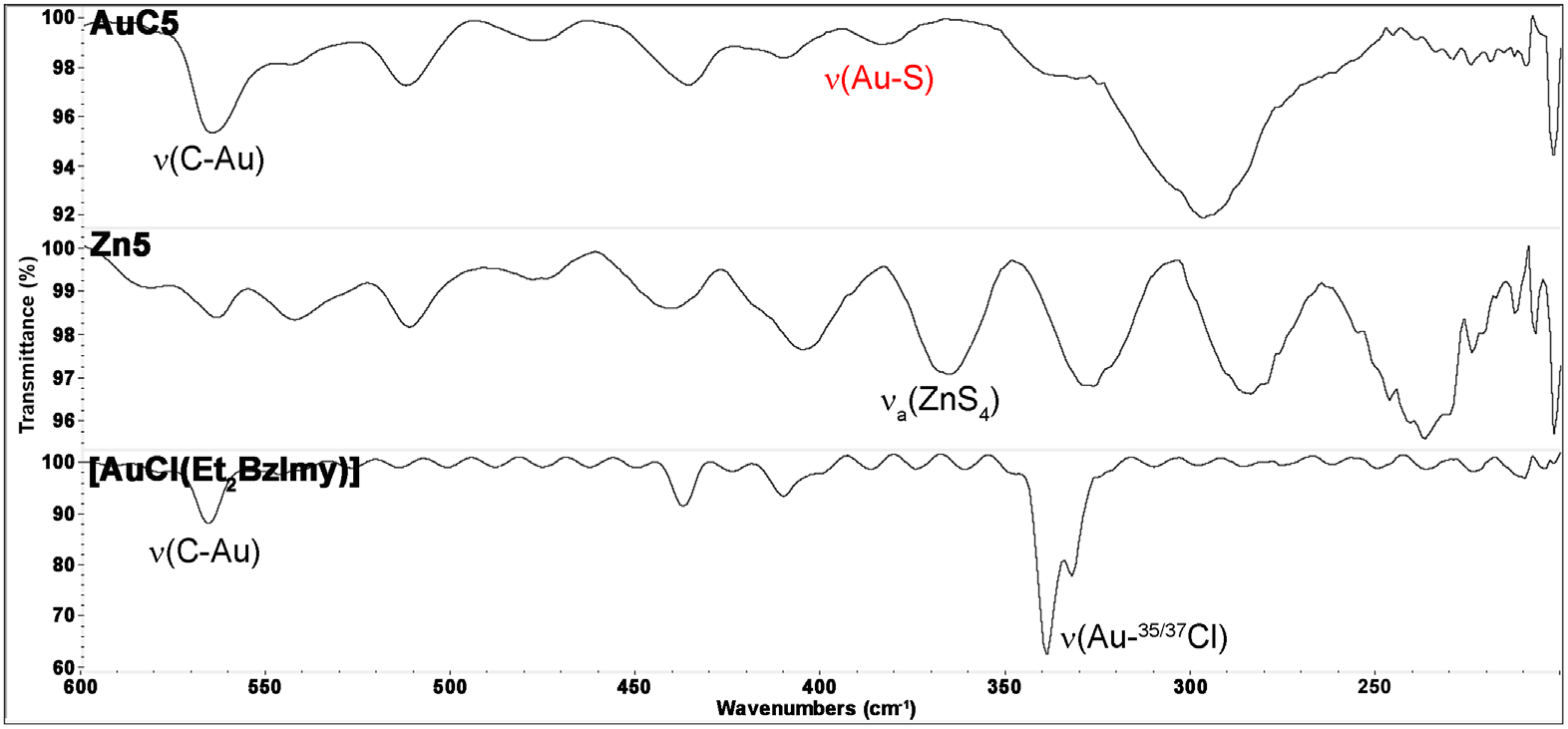
Comparison of the far‐IR spectra in CsI of [Au^I^Cl(Et_2_BzImy)], [Zn^II^(SSC‐Inp‐GlcN3)_2_] (**Zn5**), and [Au^I^(SSC‐Inp‐GlcN3)(Et_2_BzImy)] (**AuC5**).

Bands recorded in the far‐IR region also provided useful information about the metal binding modes of the ligands. Using again **AuC5** as an example (**Figure** **5**), the C‐Au stretching vibration is tentatively assigned to the band at around 565 cm^−1^ based on the little data reported in the literature for analogous scaffolds.^[^ ^52^ ^,^ ^53^ ^]^ In contrast, the disappearance of the *ν*_a_(ZnS_4_) at *ca.* 365 cm^−1^ in the spectra of the zinc(II) intermediates^[^ ^54^ ^]^ and of the *ν*(Au—Cl) at 340 cm^−1^ in the spectrum of [Au^I^Cl(Et_2_BzImy)],^[^ ^43^ ^]^ along with the appearance of a new vibration recorded for all complexes **AuC1‐5** at around 385 cm^−1^ (ascribed to the Au—S stretching),^[^ ^38^ ^]^ would indicate the successful transmetallation reaction and the consequent replacement of the chlorido ligand with one dithiocarbamato sulfur donor atom.

**Figure 6:**
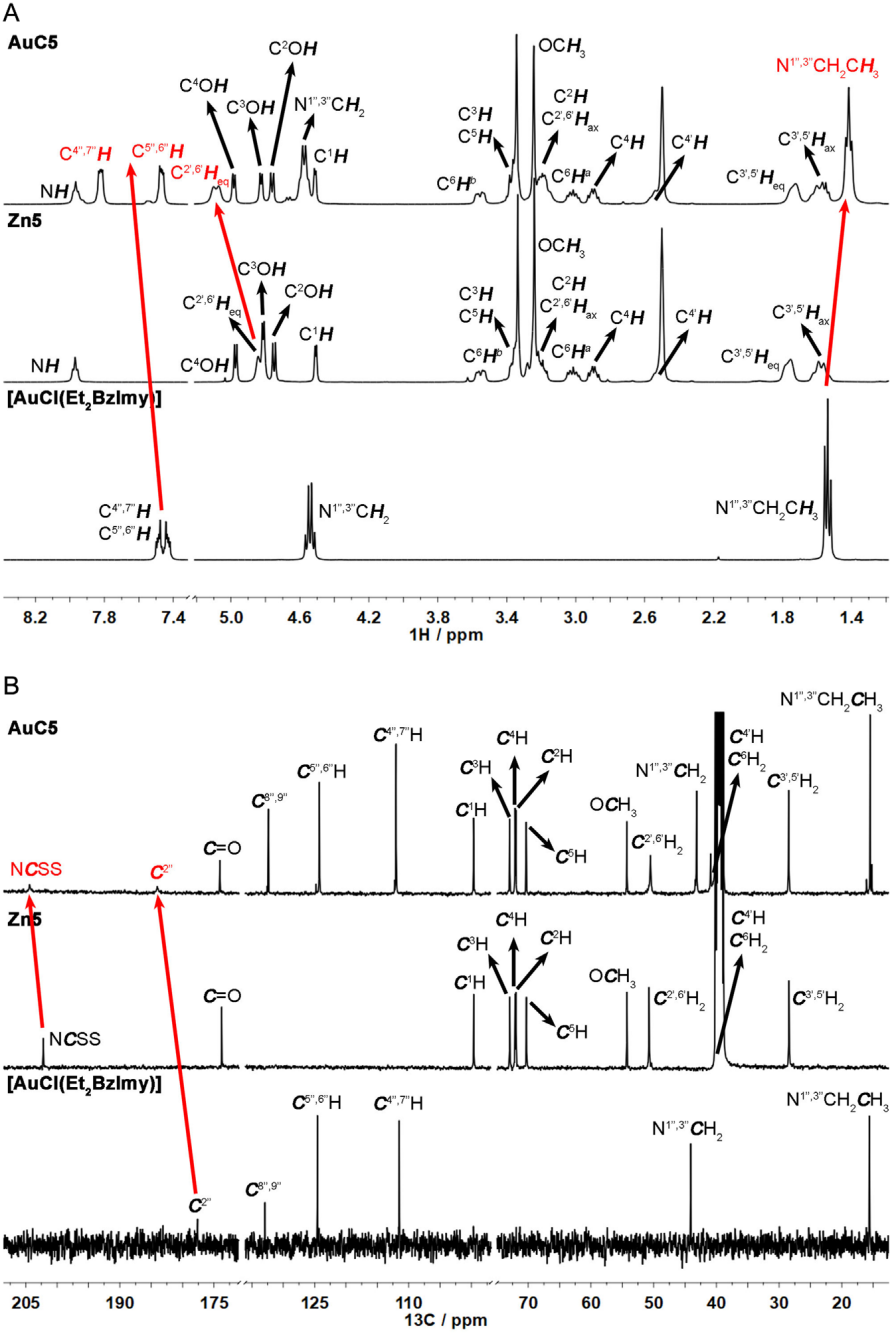
Comparison of the ^1^H A) and ^13^C{^1^H} B) NMR spectra of [Au^I^Cl(Et_2_BzImy)] in CDCl_3_, [Zn^II^(SSC‐Inp‐GlcN3)_2_] (**Zn5**) in DMSO‐D_6_, and [Au^I^(SSC‐Inp‐GlcN3)(Et_2_BzImy)] (**AuC5**) in DMSO‐D_6_.

The NMR spectra of all gold(I)‐carbene‐dithiocarbamato complexes **AuC1‐5** mostly resemble those of the zinc(II) analogs **Zn1‐5** and the gold(I) precursor [Au^I^Cl(Et_2_BzImy)] in terms of both chemical shifts and peak patterns, with very few noteworthy divergences. With reference to **Figure** **6A** as an example, the ^1^H NMR peaks of the gold(I)‐carbene complex **AuC5** are nearly superimposable to the equivalent signals of the zinc(II) intermediate **Zn5**, the only exception being a slight (0.25 ppm) downfield shift of the equatorial hydrogen atoms of the isonipecotic linker (C^2′, 6′^ *H*_eq_). As to the heterocyclic carbene moiety, compared with the [Au^I^Cl(Et_2_BzImy)] precursor a 0.12 ppm upfield shift is observed for the methyl groups of the *N*‐ethyl pendants, although the most significant deviation relates to the larger splitting between the group peaks of the aromatic hydrogens C^4",7"^H + C^5",6"^H. Similarly, no major changes were detected in the ^13^C NMR spectra (see Figure 6B as an example). The most diagnostic peak, associated with the dithiocarbamato moiety (NCSS), was consistently detected at around 204 ppm, as previously reported for this class of gold(I)‐dithiocarbamato derivatives.^[^ ^38^ ^]^ Remarkably, the peak of the quaternary carbene carbon atom (C^2"^) shifts downfield by ~6 ppm in the entire **AuC1‐5** series as compared to the [Au^I^Cl(Et_2_BzImy)] precursor, reflecting a decreased electron‐donating effect through *π*‐backdonation upon replacing the chlorido with a dithiocarbamato ligand.^[^ ^55^ ^]^

### 2.4 Crystallographic Studies

The molecular structure proposed for the gold(I) complexes here reported on the basis of the spectroscopic data was further confirmed by crystallographic studies.^[^ ^56^ ^]^ Crystals suitable for X‐ray crystallography were obtained for the non‐glycosylated gold(I)‐phosphine derivative **AuP2** and gold(I)‐carbene complexes **AuC1** and **AuC2**. Crystal data and structure refinements are summarized in Table S2 in the Supporting Information.

**Figure 7:**
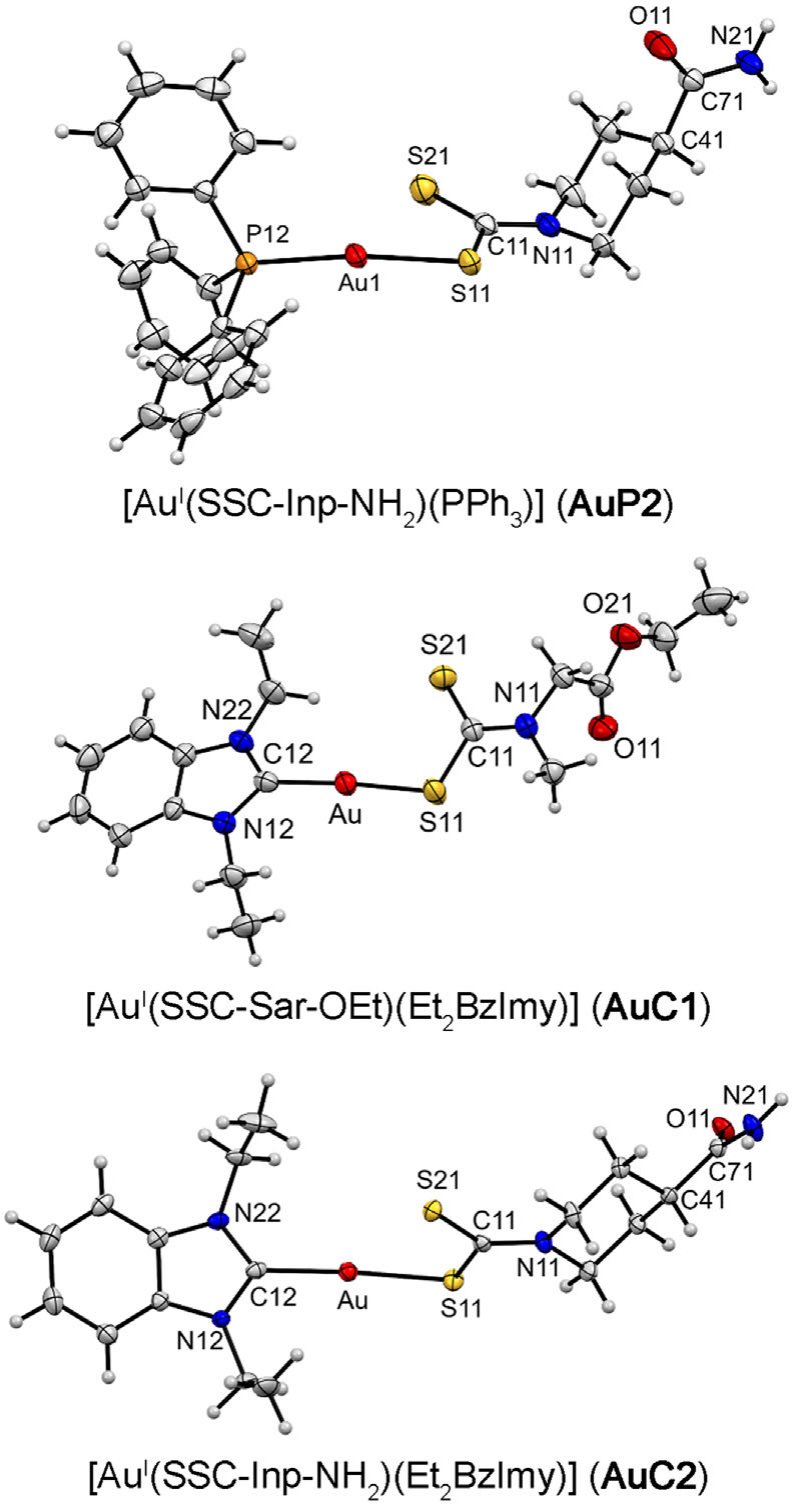
Molecular structures with atom numbering schemes of **AuP2** (CCDC 2456093, top; only one of the two molecular entities comprising the asymmetric unit is depicted for clarity), **AuC1** (CCDC 2456094, middle; only one of the two fragments of the disordered peripheral ester group is shown for clarity), and **AuC2** (CCDC 2456095, bottom). Thermal ellipsoids are depicted at the 30% probability level.

As shown in **Figure** **7**, all three complexes exhibit a gold(I) atom in a linear geometry bound by one sulfur atom of the ditiocarbamato ligand in a monodentate fashion, and by either the phosphorus atom of the triphenylphosphine ligand (**AuP2**) or the carbon atom of the 1,3‐diethylbenzimidazol‐2‐ylidene (i.e., heterocyclic carbene) ligand (**AuC1** and **AuC2**). The Au—C bond distances are 1.99(1) and 1.995(3) Å for **AuC1** and **AuC2**, respectively, and they are essentially equivalent. The Au—S bond distances vary in the range 2.288(3)−2.335(1) Å, and they are slightly longer for **AuP2**, in which the triphenylphosphine phosphorous atom is *trans* with respect to the coordinated sulfur atom. The S—Au—C and S—Au—P bonds are almost linear with angles in the range of 171–176° (see Table S3 in the Supporting Information).

The asymmetric unit of **AuP2** comprises of two molecular entities linked through two hydrogen bonds involving the peripheral amide moiety of the dithiocarbamato ligand. The same arrangement is observed for **AuC2**, in which the same type of hydrogen bonds is formed between two symmetry‐related molecules. The hydrogen bond distances in **AuP2** and **AuC2** are 2.87  (N21…O13)/2.93 (N23…O11) and 2.89 Å (N21…O11), respectively (see Figure S2 in the Supporting Information).

### 2.5 UV–Vis and Stability Studies in Solution

All the gold(III), gold(I)‐phosphine, and gold(I)‐carbene dithiocarbamato derivatives here reported were analyzed by UV–Vis spectrophotometry (see the Experimental Section for detailed assignments). Spectra were recorded at room temperature in either DMSO or DMF.

For the gold(III) derivatives two intense absorption bands were recorded at around 265 (often split in two) and 285 nm, arising from the π*←*π* intraligand transitions localized on the —NCSS moiety,^[^ ^47^ ^,^ ^57^ ^]^ along with a shoulder originated from the π*←*d* MLCT transition at ~320 nm^[^ ^58^ ^]^ and a broad very low intensity band in the range 370‐450 nm due to the spin‐allowed but symmetry‐forbidden d←d transitions localized on metal center.^[^ ^59^ ^]^

The interpretation of the UV–Vis spectra of the gold(I) complexes proved more challenging due to the presence of additional absorptions (overlapping with those localized on the ditiocarbamato group) associated with transitions specific to the phosphine or carbene ligands. For instance, the triphenylphosphine group undergoes a number of σ*←*n*, π*←*π* and π*←*n* transitions in the range 230–270 nm,^[^ ^60^ ^,^ ^61^ ^]^ and similarly does the carbene ligand Et_2_BzImy, showing three intense close π*←*π* transitions in the range 270‐290 nm.^[^ ^62^ ^]^ Finally, a characteristic band was recorded at around 300 nm for all such complexes arising from the *p*(Au)←*p*(S) LMCT transition typical of sulfur‐containing gold(I) derivatives.^[^ ^61^ ^,^ ^62^ ^]^

DMSO and DMF are solvents commonly used to prepare stock solutions of metal complexes prior to performing biological studies, especially if poorly soluble in aqueous media. All gold complexes proved stable in pure DMSO (**Au1‐5** and **AuP1‐5**) or DMF (**AuC1‐5**) over 24‐48 h, regardless of the oxidation state of the metal center and/or the type of ligands bound to it. However, to mimic a physiological‐like environment, stability studies in phosphate‐buffered saline (PBS) solution at pH 7.4 and 37°C were carried out for the various gold complexes by UV–Vis spectrophotometry.

**Figure 8:**
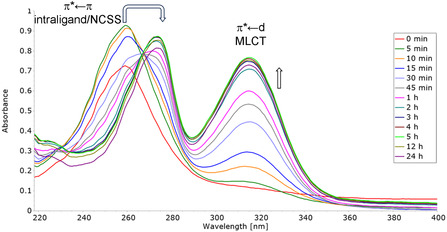
UV–vis spectra over 24 hr of **Au5** 25 μM in PBS (pH 7.4) at 37 °C.

All gold(III) derivatives **Au1‐5** behaved similarly in PBS. As an example, the UV–Vis spectra of **Au5** over time are shown in **Figure** **8**. The major spectral changes observed immediately after dissolution have been previously ascribed to the rapid hydrolysis of the gold(III) complex, resulting in the replacement of the bromide ligands by two water molecules and the subsequent formation of the di‐aquo di‐cationic species.^[^ ^63^ ^]^ However, it is well known that the hydrolyzed gold(III) species predominately bear coordinated hydroxo groups at physiological pH.^[^ ^64^ ^,^ ^65^ ^]^ In fact, the gold(III) center is strongly acidic and, thus, drastically lowers the p*K*_a_ of the coordinated water molecules, which then tend to lose one proton each eventually leading to the formation over time of the corresponding di‐hydroxo derivatives (often confirmed by the precipitation of an insoluble yellow residue identified as the [Au^III^(dithiocarbamate)(OH)_2_] species).^[^ ^63^ ^]^ This overall process induces a large sudden increase of intensity of the bands at 270 (π*←*π* intraligand transitions localized on the ‐NCSS moiety) and 320 (π*←*d* MLCT transition) nm, the former also experiencing a bathochromic shift (~15 nm) reflecting the change of coordination sphere from bromide to aquo/hydroxo ligands. After 24 hr, all absorptions tend to disappear and the solution turns colorless due to the reduction of the gold(III) species to the water‐insoluble gold(I) dimer [Au^I^_2_(dithiocarbamate)_2_] (as confirmed by the precipitation of a dark violet residue in agreement with previous findings).^[^ ^63^ ^]^

**Figure 9:**
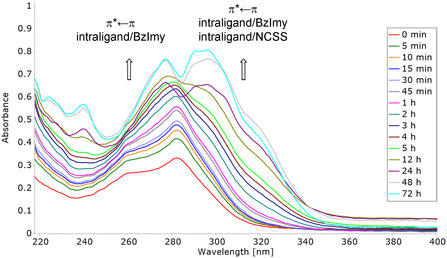
UV–vis spectra over 72 hr of **AuC5** 25 μM in PBS (pH 7.4) at 37°C.

Analogous experiments were performed for the gold(I)‐carbene counterparts **AuC1‐5** which all showed the same trend. With reference to **Figure** **9**, reporting the UV–Vis spectra of **AuC5** over time as an example, major spectral changes occur soon after dissolution as reflected by the large intensity increase and shifts of absorption maxima. Such evidence is consistent with a multistep ligand exchange reaction occurring under physiological conditions leading to the generation of the mono‐cationic bis‐carbene adduct [Au^I^(Et_2_BzImy)_2_]^+^, which was undoubtedly confirmed through separate NMR experiments (data not shown). This is in agreement with the fast ligand replacement by solvent molecules and bis‐carbene formation previously observed for other [Au^I^Cl(carbene)] complexes.^[^ ^66^ ^]^

Finally, such stability studies could not be carried out for the gold(I)‐phosphine complexes **AuP1‐5** due to their poor solubility in pure PBS.

### 2.6 Antiproliferative Activity

The capability of the gold(III) complexes **Au1**‐**5**, the gold(I)‐phosphine derivatives **AuP1‐5,** and the gold(I)‐carbene counterparts **AuC1‐5** to inhibit cell proliferation was assayed on three human tumor cell lines, namely, colorectal adenocarcinoma (HT‐29), metastatic breast adenocarcinoma (MDA‐MB‐231), and breast adenocarcinoma (MCF‐7) cells, whereas human non‐malignant kidney epithelial (RC‐124) cells were used as non‐tumor reference cell line.

Upon analyzing the overall antiproliferative data (**Table** **1**), some trends can be clearly identified. Regardless of the oxidation state of the gold center, the non‐glycosylated complexes induce a significant cytotoxic effect toward all tested tumor cell lines. Interestingly, the complexes bearing an ester moiety at the *O*‐terminus of the linker (**Au1**, **AuP1,** and **AuC1**) show greater antiproliferative activity than the analogs having a terminal primary amide (that is, **Au2**, **AuP2,** and **AuC2**), with IC_50_ values in the range 0.2‐1.4 and 1.3‐20.1 μM, respectively. This is in agreement with recent findings showing that the presence of a terminal ester (instead of amide) function enhances cytotoxicity in some gold‐dithiocarbamato complexes previously reported.^[^ ^37^ ^]^ A plausible explanation would rely on the fact that esters are generally more lipophilic than amides, and this difference in lipophilicity can affect a compound's capability to cross cell membranes and be internalized inside the cell, thus impacting its absorption, distribution, and bioavailability.^[^ ^67^ ^]^

**Table 1:** Cell growth inhibition upon incubation with the various gold(III)‐ and gold(I)‐dithiocarbamato complexes.

Complex		IC_50_ ± S.D. [μM]			
		MCF‐7	MDA‐MB‐231	HT‐29	RC‐124
**{Au** ^**III**^ **Br**_**2**_ **}**	**Au1**	0.8 ± 0.1	1.4 ± 0.2	1.2 ± 0.2	0.6 ± 0.1
	**Au2**	5.1 ± 1.0	8.9 ± 1.1	20.1 ± 0.1	9.9 ± 0.8
	**Au3**	> 50	> 50	> 50	> 50
	**Au4**	> 50	> 50	> 50	48.2 ± 13.2
	**Au5**	> 50	> 50	> 50	18.8 ± 1.7
**{Au** ^**I**^ **(PPh**_**3**_ **)}**	**AuP1**	0.2 ± 0.1	0.6 ± 0.2	0.5 ± 0.1	n.d.^a)^
	**AuP2**	1.3 ± 0.1	1.4 ± 0.3	2.9 ± 0.7	n.d.^a)^
	**AuP3**	2.6 ± 0.4	2.4 ± 0.8	8.8 ± 1.6	n.d.^a)^
	**AuP4**	2.4 ± 0.4	2.4 ± 0.7	6.3 ± 0.5	n.d.^a)^
	**AuP5**	2.5 ± 0.4	2.5 ± 0.5	6.7 ±1.1	n.d.^a)^
**{Au** ^**I**^ **(Et**_**2**_ **BzImy)}**	**AuC1**	0.5 ± 0.1	0.8 ± 0.1	1.0 ± 0.1	0.6 ± 0.1
	**AuC2**	4.9 ± 0.6	4.7 ±0.3	6.4 ± 1.3	n.d.^a)^
	**AuC3**	12.3 ± 1.4	11.5 ±0.6	22.7 ± 1.0	n.d.^a)^
	**AuC4**	10.3 ± 0.3	10.5 ± 0.4	11.3 ± 0.4	n.d.^a)^
	**AuC5**	10.6 ±0.6	9.6 ± 1.5	11.8 ± 1.1	n.d.^a)^

^a)^ n.d.: not determined.

In contrast, glycoconjugation in general did not trigger the desired cytotoxic effects, although some distinctions should be noted since the oxidation state of gold does seem to make a difference. The tumor cell growth inhibitory activity of the gold(III)‐dithiocarbamato glycoconjugate derivatives **Au3‐5** substantially decreased with respect to the non‐glycosylated gold(III) counterparts, with IC_50_ values above the highest studied dosage of 50 μM. In contrast, significant cytotoxicity was observed for the gold(I)‐glycoconjugates, but the coordination environment seems to play a major role in their biological activity. Compared with non‐glycosylated model derivatives **AuC1‐2** (whose IC_50_ values in the low micromolar range closely resemble those obtained for other mono‐carbene gold(I) complexes),^[^ ^68^ ^,^ ^69^ ^]^ the gold(I)‐carbene glycoconjugates **AuC3‐5** show slightly decreased antiproliferative effect with IC_50_ values around 10 μM in most cases. A similar trend was observed for the gold(I)‐phosphine glycoconjugates **AuP3‐5**, although in this case, IC_50_ values are closer to those of the aglycon analogs **AuP1‐2** (mostly around 2.5 μM). Remarkably, differences in the glucose‐like scaffold, such as conjugation at the C^2^ or C^6^ site of the monosaccharide, the presence of a free or *α*‐blocked (i.e., *O*‐methylated) anomeric position, do not affect the overall activity of metal‐glycoconjugates within the same set (that is, **Au3‐5**, **AuC3‐5,** and **AuP3‐5**).

Finally, antiproliferative data related to the non‐tumorigenic cell line (RC‐124) showed that no actual selectivity for tumor cells was observed for any of the complexes tested, since RC‐124 cells were affected within the same concentration range.

### 2.7 Cellular Uptake

It is often assumed that the antiproliferative activity of gold complexes is strongly linked to the efficiency of their cellular uptake and internalization. Moreover, glycoconjugates are commonly believed to enter cells through GLUTs, which, as discussed earlier, are generally over‐expressed in several cancer cells. In particular, GLUT1 is crucial for the uptake of glucose by breast cancer cells, including MDA‐MB‐231 and MCF‐7.^[^ ^70^ ^]^ Therefore, to investigate a possible relationship between cellular uptake and the in vitro antiproliferative effect of the gold(III) complexes, the cellular accumulation of the aglycon gold(III) derivative **Au2** and its glycoconjugate analog **Au4** was investigated in MCF‐7 cells. The gold levels were determined by high‐resolution continuum‐source atomic absorption spectroscopy (HR‐CS AAS) after exposure to 10 μM concentration of the selected compounds over 6 h using either a high‐glucose‐contents or glucose‐free cell culture medium. Additionally, in a view to evaluating the possible involvement of GLUTs in cell internalization, experiments were carried out also in presence of Cytochalasin B. Cytochalasin B is an endofacial GLUT1 inhibitor which disrupts the ability of GLUT1 to transport glucose, thus preventing both the net uptake of glucose and the exchange of glucose between intra‐ and extracellular compartments.^[^ ^71^ ^]^

**Figure 10:**
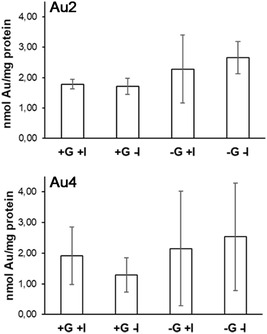
Gold content (nmol of Au per mg of cellular protein) in MCF‐7 cells incubated with 10 μM of either the model non‐glycosylated (**Au2**) or glycoconjugate (**Au4**) gold(III) complexes for 6 h. **+G**: glucose‐containing cell culture medium (4.5 g L^−1^); **‐G**: glucose‐free cell culture medium; **+I**: GLUT1 inhibitor (Cytochalasin B, 50 μM); **‐I**: no GLUT1 inhibitor added.

Results, expressed as nmol of Au per mg of cellular protein, are summarized in **Figure** **10**. Experimental data show that similar intracellular gold contents were detected for both **Au2** and **Au4** under all chosen conditions, which is in contrast with the respective antiproliferative activity (IC_50_ = 5.1 μM and > 50 1 μM, respectively). Intriguingly, a clear effect of the presence or absence of glucose in the cell culture medium or of Cytochalasin B, which would supposedly reduce GLUT1‐mediated uptake of the gold(III)‐glycoconjugate, was not observed. Therefore, based on the results obtained, the enhanced cellular uptake expected upon glyco‐functionalization of the gold(III)‐dithiocarbamato scaffold could not be confirmed. However, such effect cannot be fully excluded either since, in the experiments carried out with the metal‐glycoconjugate **Au4**, gold uptake in the glucose‐ and Cytochalasin B‐free medium was slightly higher (although not statistically significant) than under conditions where glucose, Cytochalasin B, or both were present.

Preliminary cellular uptake studies were carried out under the same experimental conditions also for the gold(I)‐carbene complexes **AuC2** (non‐glycosylated) and **AuC4** (metal‐glycoconjugate). However, such experiments proved inconclusive since the detection of intracellular gold did not return reproducible results (data not shown).

## 3 Conclusion

The goal of the research work reported here was to design three sets of gold(III)‐ and gold(I)‐based glycoconjugate anticancer agents, focusing on their potential capability to target GLUTs over‐expressed in cancer cells with a view to achieving tumor selectivity.

Three gold(III)‐dithiocarbamato glycoconjugates of the type [Au^III^Br_2_(SSC‐Inp‐GlcN)] (**Au3‐5**), their gold(I)‐phosphine counterparts [Au^I^(SSC‐Inp‐GlcN)(PPh_3_)] (**AuP3‐5**), and gold(I)‐carbene analogs [Au^I^(SSC‐Inp‐GlcN)(Et_2_BzImy)] (**AuC3‐5**) (Inp: isonipecotic moiety; GlcN: amino‐glucose scaffold; Et_2_BzImy: 1,3‐diethylbenzimidazol‐2‐ylidene moiety) have been generated, as well as the corresponding non‐glycosylated counterparts (**Au1‐2**, **AuP1‐2,** and **AuC1‐2**) bearing a terminal ester or amide function. The rationale of such designing approach was to combine in a single chemical species the well‐known anticancer activity of the {Au^III^Br_2_}, {Au^I^(PPh_3_)}, and {Au^I^ (Et_2_BzImy)} scaffolds,^[^ ^32^ ^,^ ^33^ ^,^ ^40^ ^,^ ^41^ ^]^ the intrinsic chemoprotective effect of the coordinated dithiocarbamato moiety (−NCSS),^[^ ^34^ ^]^ and the potential GLUT‐targeting properties of glucose‐like carrier substrates.^[^ ^72^ ^,^ ^73^ ^]^ The latter were selected aiming at exploring the effect of both the functionalization at different sites of the glucose moiety (C^2^ or C^6^) and the presence of a free or *α*‐methylated anomeric position.

All metal complexes were obtained in medium‐to‐high yields and high purity, and results from several analytical techniques consistently confirmed the expected formulations and molecular geometries (also supported by X‐ray crystallographic studies for the gold(I) complexes **AuP2**, **AuC1,** and **AuC2**, Figure 7). Stability studies showed that all gold complexes are stable in DMSO or DMF over 24–48 h, regardless of the oxidation state of the metal center and/or the type of coordinated ligands, whereas they undergo degradation over time in PBS solution (Figures 8 and 9).

In vitro antiproliferative assays were carried out toward three human tumor cell lines (namely, HT‐29, MDA‐MB‐231, and MCF‐7, Table 1). The model non‐glycosylated gold(III) complexes **Au1** and **Au2** returned IC_50_ values in the low micromolar range, whereas the corresponding glycoconjugates **Au3‐5** showed no cytotoxicity up to the highest tested concentration of 50 μM. On the contrary, all gold(I) derivatives proved to strongly inhibit cell proliferation with little difference (if any) between non‐glycosylated (**AuP1‐2** and **AuC1‐2**) and glycosylated (**AuP3‐5** and **AuC3‐5**) analogs, the gold(I)‐phosphine group of complexes turning up as the most active with IC_50_ values ranging from 0.2 to 8.8 μM.

A major unexpected result is the lack of consistency between antiproliferative activity and cellular accumulation in MCF‐7 cells. For example, the aglycon complex **Au2** is far more active than the corresponding gold(III)‐glycoconjugate **Au4** (IC_50_ = 5.1 μM vs > 50 μM) but returned consistently similar cellular uptake values regardless of the combination of experimental conditions such as the presence or absence of glucose in the cell culture medium and of the GLUT1 inhibitor Cytochalasin B. The connection between cytotoxicity and cellular uptake appears even more complicated to explain when considering the gold(I)‐carbene derivatives. For instance, both the aglycon complex **AuC2** and the corresponding gold(I)‐glycoconjugate **AuC4** showed significant antiproliferative activity (IC_50_ = 4.9 vs 10.3 μM) but such results did not find consistency with cell internalization data.

Altogether, experimental results allow us to speculate on the transport and antiproliferative activity mechanisms of the target gold‐glycoconjugates. Having ruled out the involvement of GLUT1 in the transport of the gold(III) derivatives inside the cell and given the generally lipophilic nature of this class of compounds,^[^ ^37^ ^]^ passive diffusion or an alternative facilitated diffusion mechanism seems plausible.^[^ ^1^ ^]^ In contrast, the lack of evidence of cellular uptake for the gold(I) species (either glycosylated or not) would suggest that such complexes are not internalized into the cells, supporting the hypothesis that they might induce cell death by acting at extracellular level, for example by interacting with the membrane lipids, proteins or other components leading to the disruption of cell membrane's functions, integrity and/or signaling pathways.^[^ ^74^ ^]^ Although such behavior has been mostly reported for gold nanoparticles,^[^ ^75^ ^]^ some gold complexes, particularly those bearing thiolato or dithiocarbamato ligands, have been shown to interact with cell membranes potentially altering their composition or damaging their structure.^[^ ^76^ ^]^

Although further in‐depth studies are required to elucidate the proposed transport and antiproliferative mechanisms and to assess additional possible biological targets, the research reported here represents a starting point for the development of gold‐based glycoconjugates as potential anticancer chemotherapeutics.

## 4 Experimental Section

4.1

4.1.1

Materials and general methods (including instrumentation used) are detailed in the Supporting Information.

##### Amino‐Sugar Precursors

2‐Amino‐2‐deoxy‐(*α*,*β*)‐d‐glucose (i.e., (*α*,*β*)‐d‐glucosamine, **GlcN1**) hydrochloride was commercially available. 1‐*O*‐Methyl‐2‐amino‐2‐deoxy‐(*α*,*β*)‐d‐glucopyranoside (**GlcN2**) and 1‐*O*‐methyl‐6‐amino‐6‐deoxy‐*α*‐d‐glucopyranoside (**GlcN3**) were synthesized, as previously described.^[^ ^38^ ^]^

##### Zinc(II) Intermediates

The zinc(II)‐dithiocarbamato intermediates [Zn^II^(SSC‐Inp‐OEt)_2_] (**Zn1**), [Zn^II^(SSC‐Inp‐NH_2_)_2_] (**Zn2**), [Zn^II^(SSC‐Inp‐GlcN1)_2_] (**Zn3**), [Zn^II^(SSC‐Inp‐GlcN2)_2_] (**Zn4**), and [Zn^II^(SSC‐Inp‐GlcN3)_2_] (**Zn5**) were prepared as previously described.^[^ ^38^ ^]^ See the Supporting Information for details.

##### Gold(III) Complexes

The gold(III)‐dithiocarbamato complexes [Au^III^Br_2_(SSC‐Inp‐OEt)] (**Au1**), [Au^III^Br_2_(SSC‐Inp‐NH_2_)] (**Au2**), [Au^III^Br_2_(SSC‐Inp‐GlcN1)] (**Au3**), [Au^III^Br_2_(SSC‐Inp‐GlcN2)] (**Au4**), and [Au^III^Br_2_(SSC‐Inp‐GlcN3)] (**Au5**) were prepared as previously described.^[^ ^38^ ^]^ See the Supporting Information for details.

##### Gold(I)‐Phosphine Complexes

The gold(I)‐phosphine‐dithiocarbamato complexes [Au^I^(SSC‐Inp‐GlcN2)(PPh_3_)] (**AuP4**) and [Au^I^(SSC‐Inp‐GlcN3)(PPh_3_)] (**AuP5**) were prepared as previously described.^[^ ^38^ ^]^ See the Supporting Information for details.

All remaining gold(I)‐phosphine‐dithiocarbamato complexes were prepared according to a modified literature procedure.^[^ ^42^ ^]^ A DMF (2 mL) solution of the appropriate zinc(II)‐dithiocarbamato intermediate (**Zn1‐3**, 1 equiv) was added dropwise to a DMF (2 mL) solution of [Au^I^Cl(PPh_3_)] (2 equiv), and the mixture was stirred at room temperature for 1 h. Addition of diethyl ether (40 mL) led to the precipitation of a yellow solid which was centrifuged, and the bulk of supernatant discarded. The remaining sticky precipitate was subsequently triturated with a 1:1 diethyl ether/dichloromethane mixture (3 × 10 mL), filtered off, and then dried under vacuum over P_2_O_5_, yielding the corresponding gold(I)‐phosphine‐dithiocarbamato derivative (**AuP1‐3**) as a yellow/pale yellow solid.

##### [Au^I^(SSC‐Inp‐OEt)(PPh_3_)] (AuP1)

Yellow solid (97.5 mg, 91% yield). M.p. 54‐56°C (dec.). Elemental analysis (%) calcd for C_27_H_29_AuNO_2_PS_2_ (MM = 691.59 g mol^−1^): C, 46.89; H, 4.23; N, 2.03; found: C, 47.08; H, 4.29; N, 2.26. FT‐IR (CsI disk, 298 K): $\bar{V}$_max_ 1729 (*ν*, C=O), 1480 (*ν*, N—CSS), 1176 (*ν*, C—OEt), 1101 (*ν*_*q*__vib_, P—Ph_3_), 1040 (*ν*, O—Et), 999/970 (*ν*, S=C—S), 710/694 (*ν*_*r*__vib_, P—Ph_3_), 540/501 (*δ*_*y*__vib_, P—Ph_3_), 510 (*ν*, C—S), 449/434 (*ν*_*t*__vib_, P—Ph_3_), 394 (*ν*, Au—P), 349 (*ν*, Au—S), 274/259 (*δ*_*x*__vib_, P—Ph_3_) cm^−1^. ^1^H NMR (400 MHz, CDCl_3_, 298 K): *δ* 7.62–7.57 (*o*‐C ***H*** Ph, m, 6H), 7.50–7.42 (*p*‐C ***H*** Ph + *m*‐C ***H*** Ph overlapped, m, 9H), 4.98 (C^2′, 6′^ ***H***_eq_, dt, ^2^ *J*_H2′/6′eq, H2′/6′ax_ = 13.3 Hz/^3^ *J*_H2′/6′eq, H3′/5_′ = 3.5 Hz, 2H), 4.15 (OC ***H***_2_, q, ^3^ *J*_H, H_ = 7.1 Hz, 2H), 3.43 (C^2′, 6′^ ***H***_ax_, td, ^2^ *J*_H2′/6′ax, H2′/6′eq_ = 12.1 Hz/^3^ *J*_H2′/6′ax, H3′/5′_ = 2.9 Hz, 2H), 2.59 (C^4′^ ***H***, tt, ^3^ *J*_H4′, H3′/5′ax_ = 10.2 Hz/^3^ *J*_H4′, H3′/5′eq_ = 4.2 Hz, 1H), 2.01 (C^3′, 5′^ ***H***_eq_, dd, ^2^ *J*_H3′/5′eq, H3′/5′ax_ = 14.1 Hz/^3^ *J*_H3′/5′eq, H2′/6_′ = 3.8 Hz, 2H), 1.89 (C^3′, 5′^ ***H***_ax_, qd, ^2^ *J*_H3′/5′ax, H3′/5′eq_ = 10.6 Hz/^3^ *J*_H3′/5′ax, H2′/6′_ = 3.8 Hz, 2H), 1.25 (C ***H***_3_, t, ^3^ *J*_H, H_ = 7.1 Hz, 3H) ppm. ^13^C{^1^H} NMR (100 MHz; CDCl_3_, 298 K): *δ* 207.0 (N ***C*** SS), 174.3 (***C*** =O), 134.3 (*o*‐***C*** H, d, ^2^ *J*_C, P_ = 13.9 Hz), 131.5 (*p*‐***C*** H, d, ^4^ *J*_C, P_ = 2.5 Hz), 130.3 (***C*** P, d, ^1^ *J*_C, P_ = 58.1 Hz), 129.2 (*m*‐***C*** H, d, ^3^ *J*_C, P_ = 11.6 Hz), 60.8 (O ***C*** H_2_), 51.0 (***C*** ^2′, 6′^H_2_), 40.6 (***C*** ^4′^H), 28.0 (***C*** ^3′, 5′^H_2_), 14.3 (***C*** H_3_) ppm. ^31^P{^1^H} NMR (162 MHz; CDCl_3_, 298 K): *δ* 36.6 (Au ***P*** Ph_3_) ppm. UV–V—is (CH_2_Cl_2_, 25 μM, 298 K): *λ*_max_ (log *ε*) 231 (4.35, σ*←*n* intraligand/PPh_3_), 236 (4.37, π*←*π* intraligand/PPh_3_), 242 (4.38, π*←*π* intraligand/PPh_3_), 269 (4.39, π*←*π* intraligand/NCSS + π*←*π* & π*←*n* intraligand/PPh_3_ overlapped), 295 (3.89, π*←*π* intraligand/NCSS), 317_sh_ (3.70, *p*(Au)←*p*(S) LMCT) nm.

##### [Au^I^(SSC‐Inp‐NH_2_)(PPh_3_)] (AuP2)

Pale yellow solid (84.5 mg, 86% yield). Yellow plate‐shaped crystals suitable for X‐ray crystallography were obtained upon slow evaporation of a dichloromethane/methanol solution of the compound (CCDC 2456093). M.p. 215‐219°C (dec.). Elemental analysis (%) calcd for C_25_H_26_AuN_2_OPS_2_ (MM = 662.56 g mol^−1^): C, 45.32; H, 3.96; N, 4.23; found: C, 46.03; H, 4.11; N, 4.82. FT‐IR (CsI disk, 298 K): $\bar{V}$_max_ 3410/3163 (*ν*_a,s_, NH_2_), 1676 (*ν*, C=O (amide I)), 1614 (*δ*_ip_, CNH_2_ (amide II)), 1477 (*ν*, N—CSS), 1101 (*ν*_*q*__vib_, P—Ph_3_), 1006/969 (*ν*, S=C‐S), 710/694 (*ν*_*r*__vib_, P—Ph_3_), 540/499 (*δ*_*y*__vib_, P—Ph_3_), 508 (*ν*, C—S), 451/430 (*ν*_*t*__vib_, P—Ph_3_), 401 (*ν*, Au—P), 358 (*ν*, Au—S), 282/254 (*δ*_*x*__vib_, P—Ph_3_) cm^−1^. ^1^H NMR (400 MHz, CDCl_3_, 298 K): *δ* 7.64–7.57 (*o*‐C ***H*** Ph, m, 6H), 7.51–7.41 (*p*‐C ***H*** Ph + *m*‐C ***H*** Ph overlapped, m, 9H), 5.55 (N ***H***_cis_, br s, 1H), 5.44 (N ***H***_trans_, br s, 1H), 5.18 (C^2′, 6′^ ***H***_eq_, dt, ^2^ *J*_H2′/6′eq, H2′/6′ax_ = 13.5 Hz/^3^ *J*_H2′/6′eq, H3′/5_′ = 3.4 Hz, 2H), 3.29 (C^2′, 6′^ ***H***_ax_, td, ^2^ *J*_H2′/6′ax, H2′/6′eq_ = 12.6 Hz/^3^ *J*_H2′/6′ax, H3′/5_′ = 3.0 Hz, 2H), 2.48 (C^4′^ ***H***, tt, ^3^ *J*_H4′, H3′/5′ax_ = 10.9 Hz/^3^ *J*_H4′, H3′/5′eq_ = 4.2 Hz, 1H), 1.99 (C^3′, 5′^ ***H***_eq_, dd, ^2^ *J*_H3′/5′eq, H3′/5′ax_ = 13.7 Hz/^3^ *J*_H3′/5′eq, H2′/6_′ = 3.6 Hz, 2H), 1.86 (C^3′, 5′^ ***H***_ax_, qd, ^2^ *J*_H3′/5′ax, H3′/5′eq_ = 11.2 Hz/^3^ *J*_H3′/5′ax, H2′/6_′ = 3.9 Hz, 2H) ppm. ^13^C{^1^H} NMR (100 MHz; CDCl_3_, 298 K): *δ* 207.2 (N ***C*** SS), 176.3 (***C*** =O), 134.3 (*o*‐***C*** H, d, ^2^ *J*_C, P_ = 14.1 Hz), 131.5 (*p*‐***C*** H, d, ^4^ *J*_C, P_ = 1.2 Hz), 130.3 (***C*** P, d, ^1^ *J*_C, P_ = 56.9 Hz), 129.2 (*m*‐***C*** H, d, ^3^ *J*_C, P_ = 11.5 Hz), 51.0 (***C*** ^2′, 6′^H_2_), 41.9 (***C*** ^4′^H), 28.7 (***C*** ^3′, 5′^H_2_) ppm. ^31^P{^1^H} NMR (162 MHz; CDCl_3_, 298 K): *δ* 36.6 (Au ***P*** Ph_3_) ppm. UV–Vis (CH_2_Cl_2_, 25 μM, 298 K): *λ*_max_ (log *ε*) 232 (4.41, σ*←*n* intraligand/PPh_3_), 235 (4.42, π*←*π* intraligand/PPh_3_), 242 (4.43, π*←*π* intraligand/PPh_3_), 269 (4.45, π*←*π* intraligand/NCSS + π*←*π* & π*←*n* intraligand/PPh_3_ overlapped), 297 (3.96, π*←*π* intraligand/NCSS), 316_sh_ (3.80, *p*(Au)←*p*(S) LMCT) nm.

##### [Au^I^(SSC‐Inp‐GlcN1)(PPh_3_)] (AuP3)

Pale yellow solid (85.3 mg, 68% yield). M.p. 170‐172°C (dec.). Elemental analysis (%) calcd for C_31_H_36_AuN_2_O_6_PS_2_ (MM = 824.70 g mol^−1^): C, 45.15; H, 4.40; N, 3.40; found: C, 44.89; H, 4.39; N, 3.54. FT‐IR (CsI disk, 298 K): $\bar{V}$_max_ 3401 (*ν*, OH + NH overlapped), 1650 (*ν*, C=O (amide I)), 1544 (*δ*_ip_, CNH (amide II)), 1482 (*ν*, N—CSS), 1101 (*ν*_*q*__vib_, P—Ph_3_), 1060 (*ν*, C—OH), 999/955 (*ν*, S=C—S), 711/693 (*ν*_*r*__vib_, P—Ph_3_), 539/500 (*δ*_*y*__vib_, P—Ph_3_), 510 (*ν*, C—S), 446/426 (*ν*_*t*__vib_, P—Ph_3_), 397 (*ν*, Au—P), 369 (*ν*, Au—S), 280/250 (*δ*_*x*__vib_, P—‐Ph_3_) cm^−1^. ^1^H NMR (400 MHz, DMSO‐D_6_, 298 K): *δ* 7.70 (N ***H*** *β*, d, ^3^ *J*_NH,H2_ = 8.6 Hz, 0.3H), 7.66 (N ***H*** *α*, d, ^3^ *J*_NH,H2_ = 7.9 Hz, 1H), 7.63–7.55 (C ***H*** Ph *α* and *β* overlapped, m, 19.5H), 6.50 (C^1^O ***H*** *β*, d, ^3^ *J*_OH1,H1_ = 6.4 Hz, 0.3H), 6.43 (C^1^O ***H*** *α*, d, ^3^ *J*_OH1,H1_ = 4.5 Hz, 1H), 4.95–4.86 (C^3^O ***H*** *α* + C^1^ ***H*** *α* + C^2′, 6′^ ***H***_eq_ *α* and *β* + C^4^O ***H*** *β* overlapped, m, 4.9H), 4.82 (C^3^O ***H*** *β*, d, ^3^ *J*_OH3,H3_ = 5.4 Hz, 0.3H), 4.61 (C^4^O ***H*** *α*, d, ^3^ *J*_OH4,H4_ = 5.6 Hz, 1H), 4.54 (C^6^O ***H*** *β*, t, ^3^ *J*_OH6,H6(dia)_ = 5.8 Hz, 0.3H), 4.45 (C^1^ ***H*** *β*, t, ^3^ *J*_H1,H2_ = 6.0 Hz, 0.3H), 4.44 (C^6^O ***H*** *α*, t, ^3^ *J*_OH6,H6(dia)_ = 6.1 Hz, 1H), 3.70–3.39 (C^2^ ***H*** *α* + C^4^ ***H*** *α* + C^5^ ***H*** *α* + C^6^ ***H***_2_ *α* and *β* overlapped, m, 5.6H), 3.33–3.20 (C^2′, 6′^ ***H***_ax_ *α* and *β* + C^2^ ***H*** *β* + C^3^ ***H*** *β* overlapped, m, 3.2H), 3.14–3.03 (C^3^ ***H*** *α* + C^4^ ***H*** *β* + C^5^ ***H*** *β* overlapped, m, 1.6H), 2.58 (C^4′^ ***H*** *α*, tt, ^3^ *J*_H4′,H3′/5′ax_ = 14.6 Hz/^3^ *J*_H4′,H3′/5′eq_ = 4.2 Hz, 1H), 2.4 (C^4′^ ***H*** *β*, tt, ^3^ *J*_H4′,H3′/5′ax_ = 11.0 Hz/^3^ *J*_H4′,H3′/5′eq_ = 4.2 Hz, 0.3H), 1.78 (C^3′, 5′^ ***H***_eq_ *α* and *β* overlapped, br t, ^2^ *J*_H3′/5′eq,H3′/5′ax_ = 11.5 Hz, 2.6H), 1.61 (C^3′, 5′^ ***H***_ax_ *α* and *β* overlapped, br t, ^2^ *J*_H3′/5′ax,H3′/5′eq_ = 11.3 Hz, 2.6H) ppm. ^13^C{^1^H} NMR (100 MHz; DMSO‐D_6_, 298 K): *δ* 204.39 (N ***C*** SS *β*), 204.37 (N ***C*** SS *α*), 173.9 (***C*** =O *α* and *β* overlapped), 133.7 (*o*‐***C*** H *α* and *β* overlapped, d, ^2^ *J*_C,P_ = 13.9 Hz), 131.9 (*p*‐***C*** H *α* and *β* overlapped, br s, ^4^ *J*_C,P_ = not detectable), 130.1 (***C*** P *α* and *β* overlapped, d, ^1^ *J*_C,P_ = not detectable due to overlapping), 129.5 (*m*‐***C*** H *α* and *β* overlapped, d, ^3^ *J*_C,P_ = 11.2 Hz), 95.4 (***C*** ^1^H *β*), 90.5 (***C*** ^1^H *α*), 76.9 (***C*** ^5^H *β*), 74.2 (***C*** ^3^H *β*), 72.1 (***C*** ^5^H *α*), 71.1 (***C*** ^3^H *α*), 70.8 (***C*** ^4^H *β*), 70.4 (***C*** ^4^H *α*), 61.2 (***C*** ^6^H_2_ *β*), 61.1 (***C*** ^6^H_2_ *α*), 57.0 (***C*** ^2^H *β*), 54.3 (***C*** ^2^H *α*), 50.7 (***C*** ^2′, 6′^H_2_ *α* and *β* overlapped), 41.0 (***C*** ^4′^H *β*), 40.5 (***C*** ^4′^H *α*), 28.54 (***C*** ^3′, 5′^H_2_ *β*), 28.45 (***C*** ^3′, 5′^H_2_ *α*) ppm. ^31^P{^1^H} NMR (162 MHz; DMSO‐D_6_, 298 K): *δ* 36.4 (Au ***P*** Ph_3_) ppm. Solution *α*:*β* anomers ratio ≈3.3:1 (based on the ^1^H NMR spectrum). UV–Vis (DMSO, 25 μM, 298 K): *λ*_max_ (log *ε*) 272 (4.29, π*←*π* intraligand/NCSS + π*←*π* & π*←*n* intraligand/PPh_3_ overlapped), 296 (3.93, π*←*π* intraligand/NCSS), 319_sh_ (3.66, *p*(Au)←*p*(S) LMCT) nm.

##### Gold(I)‐Carbene Complexes

The synthesis and characterization of the gold(I)‐carbene precursor [Au^I^Cl(Et_2_BzImy)] (prepared as previously reported in the literature)^[^ ^69^ ^]^ and the ligand (PPh_4_)(SSC‐Sar‐OEt) are detailed in the Supporting Information.

[Au^I^(SSC‐Sar‐OEt)(Et_2_BzImy)] (**AuC1**): A 6:1 water/acetone (10 mL) solution of (PPh_4_)(SSC‐Sar‐OEt) (119.0 mg, 0.22 mmol) was added dropwise to an acetone (2 mL) solution of [Au^I^Cl(Et_2_BzImy)] (AuC0, 84.8 mg, 0.21 mmol). The mixture was stirred at room temperature for 1 h, leading to the precipitation of an off‐white solid. The precipitate was then centrifuged, and the bulk of supernatants was discarded. The solid residue was subsequently washed with water (3 × 15 mL), filtered off, and then dried under vacuum over P_2_O_5_, yielding the title compound as an off‐white solid.

Off‐white solid (114.3 mg, 97% yield). Colorless needle‐shaped crystals suitable for X‐ray crystallography were obtained upon slow evaporation of a DMF solution of the compound (CCDC 2456094). M.p. 144‐146°C (dec.). Elemental analysis (%) calcd for C_17_H_24_AuN_3_O_2_S_2_ (MM = 563.48 g mol^−1^): C, 36.24; H, 4.29; N, 7.46; found: C, 36.31; H, 4.23; N, 7.42. FT‐IR (CsI disk, 298 K): $\bar{V}$_max_ 1739 (*ν*, C=O), 1483 (*ν*, N—CSS), 1461 (*ν*, C=N), 1197 (*ν*, C—OEt), 1088 (*ν*, C—N), 1042 (*ν*, N—Et), 1030 (*ν*, O—Et), 1001/982 (*ν*, S=C—S), 569 (*ν*, C—Au), 496 (*ν*, C—S), 346 (*ν*, Au—S) cm^−1^. ^1^H NMR (400 MHz, CDCl_3_, 298 K): *δ* 7.48–7.39 (C^4",7"^ ***H*** + C^5",6"^ ***H*** overlapped, m, 4H), 4.81 (NC ***H***_2_, s, 2H), 4.54 (N^1",3"^C ***H***_2_, q, ^3^ *J*_H,H_ = 7.2 Hz, 4H), 4.24 (OC ***H***_2_, q, ^3^ *J*_H,H_ = 7.1 Hz, 2H), 3.58 (NC ***H***_3_, s, 3H), 1.54 (N^1",3"^CH_2_C ***H***_3_, t, ^3^ *J*_H,H_ = 7.2 Hz, 6H), 1.30 (C ***H***_3_, t, ^3^ *J*_H,H_ = 7.1 Hz, 3H) ppm. ^13^C{^1^H} NMR (100 MHz; CDCl_3_, 298 K): *δ* 217.7 (N ***C*** SS), 185.6 (***C*** ^2"^), 168.4 (***C*** =O), 133.1 (***C*** ^8",9"^), 124.2 (***C*** ^5",6"^H), 111.4 (***C*** ^4",7"^H), 61.4 (O ***C*** ^’^H_2_), 58.3 (N ***C*** ^’^H_2_), 44.5 (N ***C*** ^’^H_3_), 43.9 (N^1",3"^ ***C*** H_2_), 15.6 (N^1",3"^CH_2_ ***C*** H_3_), 14.4 (***C*** ^’^H_3_) ppm. UV–V—is (DMF, 20 μM, 298 K): *λ*_max_ (log *ε*) 271 (4.32, π*←*π* intraligand/BzImy), 279 (4.40, π*←*π* intraligand/BzImy), 286 (4.52, π*←*π* intraligand/NCSS + π*←*π* intraligand/BzImy overlapped), 304 (4.09, *p*(Au)←*p*(S) LMCT) nm.

All remaining gold(I)‐carbene‐dithiocarbamato complexes were prepared as follows. A DMF (2 mL) solution of the appropriate zinc(II)‐dithiocarbamato intermediate (**Zn2‐Zn5**, 1 equiv) was added dropwise to a DMF (2 mL) solution of [Au^I^Cl(Et_2_BzImy)] (2 equiv), and the mixture was stirred at room temperature for 5 min. Addition of diethyl ether (60 mL) led to the precipitation of a yellow solid, which was centrifuged, and the bulk of supernatant discarded. The precipitate was subsequently washed with diethyl ether (3 × 10 mL), dichloromethane (3 × 10 mL), filtered off, and then dried under vacuum over P_2_O_5_, yielding the corresponding gold(I)‐carbene dithiocarbamato derivative (**AuC2‐AuC5**) as an off‐white/pale yellow solid.

##### [Au^I^(SSC‐Inp‐NH_2_)(Et_2_BzImy)] (AuC2)

Off‐white solid (76.0 mg, 83% yield). Colorless needle‐shaped crystals suitable for X‐ray crystallography were obtained upon slow evaporation of a DMF solution of the compound (CCDC 2456095). M.p. 202‐219°C (dec.). Elemental analysis (%) calcd for C_18_H_25_AuN_4_OS_2_ (MM = 574.51 g mol^−1^): C, 37.63; H, 4.39; N, 9.75; found: C, 37.68; H, 4.61; N, 9.75. FT‐IR (CsI disk, 298 K): $\bar{V}$_max_ 3443/3160 (*ν*_a,s_, NH_2_), 1685 (*ν*, C=O (amide I)), 1626 (*δ*_ip_, CNH_2_ (amide II)), 1477 (*ν*, N—CSS), 1463 (*ν*, C=N), 1087 (*ν*, C—N), 1039 (*ν*, N—Et), 1004/964 (*ν*, S=C—S), 559 (*ν*, C—Au), 509 (*ν*, C—S), 384 (*ν*, Au—S) cm^−1^. ^1^H NMR (400 MHz, DMSO‐D_6_, 298 K): *δ* 7.84–7.80 (C^4",7"^ ***H***, m, 2H), 7.49–7.45 (C^5",6"^ ***H***, m, 2H), 7.35 (N ***H***_cis_, br s, 1H), 6.86 (N ***H***_trans_, br s, 1H), 5.08 (C^2′, 6′^ ***H***_eq_, br d, ^2^ *J*_H2′/6′eq,H2′/6′ax_ = 12.8 Hz, 2H), 4.58 (N^1",3"^C ***H***_2_, q, ^3^ *J*_H,H_ = 7.1 Hz, 4H), 3.19 (C^2′, 6′^ ***H***_ax_, br t, ^2^ *J*_H2′/6′ax,H2′/6′eq_ = 11.4 Hz, 2H), 2.43 (C^4′^ ***H***, tt, ^3^ *J*_H4′,H3′/5′ax_ = 11.0 Hz/^3^ *J*_H4′,H3′/5′eq_ = 3.9 Hz, 1H), 1.78 (C^3′, 5′^ ***H***_eq_, dd, ^2^ *J*_H3′/5′eq,H3′/5′ax_ = 12.7 Hz/^3^ *J*_H3′/5′eq,H2′/6_′ = 2.6 Hz, 2H), 1.56 (C^3′, 5′^ ***H***_ax_, qd, ^2^ *J*_H3′/5′ax,H3′/5′eq_ = 12.2 Hz/^3^ *J*_H3′/5′ax,H2′/6_′ = 3.2 Hz, 2H), 1.42 (N^1",3"^CH_2_C ***H***_3_, t, ^3^ *J*_H,H_ = 7.1 Hz, 6H) ppm. ^13^C{^1^H} NMR (100 MHz; DMSO‐D_6_, 298 K): *δ* 204.5 (N ***C*** SS), 184.2 (***C*** ^2"^), 175.9 (***C*** =O), 132.4 (***C*** ^8",9"^),124.2 (***C*** ^5",6"^H), 112.0 (***C*** ^4",7"^H), 50.4 (***C*** ^2′, 6′^H_2_), 43.1 (N^1",3"^ ***C*** H_2_), 40.9 (***C*** ^4′^H), 28.4 (***C*** ^3′, 5′^H_2_), 15.5 (N^1",3"^CH_2_ ***C*** H_3_) ppm. UV–Vis (DMF, 20 μM, 298 K): *λ*_max_ (log *ε*) 272_sh_ (4.35, π*←*π* intraligand/BzImy), 281_sh_ (4.50, π*←*π* intraligand/BzImy), 288 (4.60, π*←*π* intraligand/NCSS + π*←*π* intraligand/BzImy overlapped), 305 (4.12, *p*(Au)←*p*(S) LMCT) nm.

##### [Au^I^(SSC‐Inp‐GlcN1)(Et_2_BzImy)] (AuC3)

Pale yellow solid (80.2 mg, 88% yield). M.p. 154‐157°C (dec.). Elemental analysis (%) calcd for C_24_H_35_AuN_4_O_6_S_2_ (MM = 736.65 g mol^−1^): C, 39.13; H, 4.79; N, 7.61; found: C, 39.01; H, 4.65; N, 7.77 FT‐IR (CsI disk, 298 K): $\bar{V}$_max_ 3401 (*ν*, OH + NH overlapped), 1657 (*ν*, C=O (amide I)), 1543 (*δ*_ip_, CNH (amide II)), 1490 (*ν*, N—CSS), 1463 (*ν*, C=N), 1089 (*ν*, C—N), 1059 (*ν*, C—OH), 1041 (*ν*, N—Et), 998/954 (*ν*, S=C—S), 567 (*ν*, C—Au), 507 (*ν*, C—S), 385 (*ν*, Au—S) cm^−1^. ^1^H NMR (400 MHz, DMSO‐D_6_, 298 K): *δ* 7.94–7.93 (C^4",7"^ ***H*** *β*, m, 0.8H), 7.83–7.82 (C^4",7"^ ***H*** *α*, m, 2H), 7.68 (N ***H*** *β*, d, ^3^ *J*_NH,H2_ = 8.4 Hz, 0.4H), 7.62 (N ***H*** *α*, d, ^3^ *J*_NH,H2_ = 6.9 Hz, 1H), 6.48 (C^1^O ***H*** *β*, d, ^3^ *J*_OH1,H1_ = 6.4 Hz, 0.4H), 6.41 (C^1^O ***H*** *α*, d, ^3^ *J*_OH1,H1_ = 4.3 Hz, 1H), 7.56–7.54 (C^5",6"^ ***H*** *β*, m, 0.8H), 7.48–7.46 (C^5",6"^ ***H*** *α*, m, 2H), 5.07 (C^2′, 6′^ ***H***_eq_ *α*, br s, 2H), 4.93–4.92 (C^1^ ***H*** *α* + C^2′, 6′^ ***H***_eq_ *β* + C^4^O ***H*** *β* overlapped, m, 2.2H), 4.90 (C^3^O ***H*** *α*, d, ^3^ *J*_OH3,H3_ = 5.4 Hz, 1H), 4.80 (C^3^O ***H*** *β*, d, ^3^ *J*_OH3,H3_ = 5.4 Hz, 0.4H), 4.67 (N^1",3"^C ***H***_2_ *β*, q, ^3^ *J*_H,H_ = 7.2 Hz, 0.8H), 4.60–4.57 (N^1",3"^C ***H***_2_ *α* + C^4^O ***H*** a overlapped, m, 5H), 4.52 (C^6^O ***H*** *β*, t, ^3^ *J*_OH6,H6(dia)_ = 5.9 Hz, 0.4H), 4.46 (C^1^ ***H*** *β*, t, ^3^ *J*_H1,H2_ = 7.6 Hz, 0.4H), 4.42 (C^6^O ***H*** *α*, t, ^3^ *J*_OH6,H6(dia)_ = 5.8 Hz, 1H), 3.69–3.41 (C^2^ ***H*** *α* + C^4^ ***H*** *α* + C^5^ ***H*** *α* + C^6^ ***H***_2_ *α* and *β* overlapped, m, 5.8H), 3.29–3.19 (C^2′, 6′^ ***H***_ax_ *α* and *β* + C^2^ ***H*** *β* + C^3^ ***H*** *β* overlapped, m, 3.6H), 3.14–3.02 (C^3^ ***H*** *α* + C^4^ ***H*** *β* + C^5^ ***H*** *β* overlapped, m, 1.8H), 2.61–2.54 (C^4′^ ***H*** *α*, br m, 1H), 2.39–2.38 (C^4′^ ***H*** *β*, br m, 0.4H), 1.82–1.70 (C^3′, 5′^ ***H***_eq_ *α* and *β* overlapped, br m, 2.8H), 1.65–1.57 (C^3′, 5′^ ***H***_ax_ *α* and *β* overlapped, br m, 2.8H), 1.56 (N^1",3"^CH_2_C ***H***_3_ *β*, t, ^3^ *J*_H,H_ = 7.2 Hz, 2.4H), 1.43 (N^1",3"^CH_2_C ***H***_3_ *α*, t, ^3^ *J*_H,H_ = 7.1 Hz, 6H) ppm. ^13^C{^1^H} NMR (100 MHz; DMSO‐D_6_, 298 K): *δ* 204.4 (N ***C*** SS *α* and *β* overlapped), 189.2 (***C*** ^2"^ *α*), 184.2 (***C*** ^2"^ *β*), 173.9 (***C*** =O *α* and *β* overlapped), 132.5 (***C*** ^8",9"^ *β*), 132.4 (***C*** ^8",9"^ *α*), 124.8 (***C*** ^5",6"^H *β*), 124.2 (***C*** ^5",6"^H *α*), 112.2 (***C*** ^4",7"^H *β*), 112.0 (***C*** ^4",7"^H *α*), 95.4 (***C*** ^1^H *β*), 90.5 (***C*** ^1^H *α*),76.9 (***C*** ^5^H *β*), 74.2 (***C*** ^3^H *β*), 72.1 (***C*** ^5^H *α*), 71.1 (***C*** ^3^H *α*), 70.8 (***C*** ^4^H *β*), 70.4 (***C*** ^4^H *α*),61.2 (***C*** ^6^H_2_ *β*), 61.1 (***C*** ^6^H_2_ *α*),57.0 (***C*** ^2^H *β*), 54.3 (***C*** ^2^H *α*), 50.5 (***C*** ^2′, 6′^H_2_ *α* and *β* overlapped), 43.4 (N^1",3"^ ***C*** H_2_ *β*), 43.1 (N^1",3"^ ***C*** H_2_ *α*), 40.8 (***C*** ^4′^H *β*), 40.1 (***C*** ^4′^H *α*), 28.5 (***C*** ^3′, 5′^H_2_ *β*), 28.4 (***C*** ^3′, 5′^H_2_ *α*), 16.1 (N^1",3"^CH_2_ ***C*** H_3_ *β*), 15.5 (N^1",3"^CH_2_ ***C*** H_3_ *α*) ppm. Solution *α*:*β* anomers ratio ≈2.5:1 (based on the ^1^H NMR spectrum). UV–Vis (DMF, 20 μM, 298 K): *λ*_max_ (log *ε*) 272_sh_ (4.47, π*←*π* intraligand/BzImy), 280_sh_ (4.59, π*←*π* intraligand/BzImy), 287 (4.70, π*←*π* intraligand/NCSS + π*←*π* intraligand/BzImy overlapped), 303 (4.24, *p*(Au)←*p*(S) LMCT) nm.

##### [Au^I^(SSC‐Inp‐GlcN2)(Et_2_BzImy)] (AuC4)

Pale yellow solid (83.5 mg, 88% yield). M.p. 155‐157°C (dec.). Elemental analysis (%) calcd for C_25_H_37_AuN_4_O_6_S_2_ (MM = 750.68 g mol^−1^): C, 40.00; H, 4.97; N, 7.46; found: C, 40.23; H, 4.96; N, 7.27 FT‐IR (CsI disk, 298 K): $\bar{V}$_max_ 3402 (*ν*, OH + NH overlapped), 1658 (*ν*, C=O (amide I)), 1542 (*δ*_ip_, CNH (amide II)), 1491 (*ν*, N—CSS), 1464 (*ν*, C=N), 1089 (*ν*, C—N), 1055/1043 (*ν*, C—OH + C^1^‐O‐CH_3_ overlapped), 1042 (*ν*, N—Et), 1000/949 (*ν*, S=C—S), 568 (*ν*, C—Au), 504 (*ν*, C—S), 384 (*ν*, Au—S) cm^−1^. ^1^H NMR (400 MHz, DMSO‐D_6_, 298 K): *δ* 7.84–7.81 (C^4",7"^ ***H***, m, 2H), 7.78 (N ***H***, d, ^3^ *J*_NH,H2_ = 8.1 Hz, 1H), 7.49–7.46 (C^5",6"^ ***H***, m, 2H), 5.09 (C^2′, 6′^ ***H***_eq_, br d, ^2^ *J*_H2′/6′eq,H2′/6′ax_ = 12.4 Hz, 2H), 5.01 (C^4^O ***H***, d, ^3^ *J*_OH4,H4_ = 5.6 Hz, 1H), 4.73 (C^3^O ***H***, d, ^3^ *J*_OH3,H3_ = 5.9 Hz, 2H), 4.58 (N^1",3"^C ***H***_2_, q, ^3^ *J*_H,H_ = 7.1 Hz, 4H), 4.55 (C^6^O ***H***, t, ^3^ *J*_OH6,H6(dia)_ = 6.1 Hz, 1H), 4.54 (C^1^ ***H***, d, ^3^ *J*_H1,H2_ = 3.7 Hz, 1H), 3.68–3.62 (C^2^ ***H*** + C^6^ ***H*** ^b^ overlapped, m, 2H), 3.49–3.43 (C^3^ ***H*** + C^6^ ***H*** ^a^ overlapped, m, 2H), 3.33–3.27 (C^5^ ***H***, m, 1H), 3.24 (OC ***H***_3_, s, 3H), 3.15–3.09 (C^4^ ***H*** + C^2′, 6′^ ***H***_ax_ overlapped, m, 3H), 2.58 (C^4′^ ***H*** a, tt, ^3^ *J*_H4′,H3′/5′ax_ = 11.1 Hz/^3^ *J*_H4′,H3′/5′eq_ = 3.7 Hz, 1H), 1.76 (C^3′, 5′^ ***H***_eq_, br t, ^2^ *J*_H3′/5′eq,H3′/5′ax_ = 10.8 Hz, 2H), 1.59 (C^3′, 5′^ ***H***_ax_, br q, ^2^ *J*_H3′/5′ax,H3′/5′eq_ = 11.1 Hz, 2H), 1.42 (N^1",3"^CH_2_C ***H***_3_, t, ^3^ *J*_H,H_ = 7.1 Hz, 6H) ppm. ^13^C{^1^H} NMR (100 MHz; DMSO‐D_6_, 298 K): *δ* 204.5 (N ***C*** SS), 185.0 (***C*** ^2"^), 174.1 (***C*** =O), 132.4 (***C*** ^8",9"^), 124.2 (***C*** ^5",6"^H), 112.0 (***C*** ^4",7"^H), 97.9 (***C*** ^1^H), 72.8 (***C*** ^5^H), 70.8 (***C*** ^3^H), 70.7 (***C*** ^4^H), 60.8 (***C*** ^6^H_2_), 54.5 (O ***C*** H_3_), 53.7 (***C*** ^2^H), 50.5 (***C*** ^2′, 6′^H_2_), 43.1 (N^1",3"^ ***C*** H_2_), 40.8 (***C*** ^4′^H), 28.5 (***C*** ^3′, 5′^H_2_), 15.5 (N^1",3"^CH_2_ ***C*** H_3_) ppm. No NMR signals undoubtedly assignable to the *β* anomer were detected. UV–Vis (DMF, 20 μM, 298 K): *λ*_max_ (log *ε*) 272_sh_ (4.33, π*←*π* intraligand/BzImy), 281_sh_ (4.48, π*←*π* intraligand/BzImy), 287 (4.58, π*←*π* intraligand/NCSS + π*←*π* intraligand/BzImy overlapped), 305 (4.10, *p*(Au)←*p*(S) LMCT) nm.

##### [Au^I^(SSC‐Inp‐GlcN3)(Et_2_BzImy)] (AuC5)

Pale yellow solid (82.8 mg, 88% yield). M.p. 151‐155°C (dec.). Elemental analysis (%) calcd for C_25_H_37_AuN_4_O_6_S_2_ (MM = 750.68 g mol^−1^): C, 40.00; H, 4.97; N, 7.46; found: C, 39.76; H, 4.92; N, 7.68 FT‐IR (CsI disk, 298 K): $\bar{V}$_max_ 3401 (*ν*, OH + NH overlapped), 1656 (*ν*, C=O (amide I)), 1545 (*δ*_ip_, CNH (amide II)), 1492 (*ν*, N—CSS), 1464 (*ν*, C=N), 1088 (*ν*, C—N), 1051 (*ν*, C—OH + C^1^—O—CH_3_ + N—Et overlapped), 1007/956 (*ν*, S=C—S), 565 (*ν*, C—Au), 513 (*ν*, C—S), 384 (*ν*, Au—S) cm^−1^. ^1^H NMR (400 MHz, DMSO‐D_6_, 298 K): *δ* 7.97 (N ***H***, t, ^3^ *J*_NH,H6(dia)_ = 5.7 Hz, 1H), 7.83–7.81 (C^4",7"^ ***H***, m, 2H), 7.48–7.46 (C^5",6"^ ***H***, m, 2H), 5.09 (C^2′, 6′^ ***H***_eq_, br d, ^2^ *J*_H2′/6′eq,H2′/6′ax_ = 11.6 Hz, 2H), 4.98 (C^4^O ***H***, d, ^3^ *J*_OH4,H4_ = 5.4 Hz, 1H), 4.82 (C^3^O ***H***, d, ^3^ *J*_OH3,H3_ = 4.8 Hz, 1H), 4.76 (C^2^O ***H***, d, ^3^ *J*_OH2,H2_ = 6.4 Hz, 1H), 4.58 (N^1",3"^C ***H***_2_, q, ^3^ *J*_H,H_ = 6.9 Hz, 4H), 4.51 (C^1^ ***H***, d, ^3^ *J*_H1,H2_ = 3.4 Hz, 1H), 3.55 (C^6^ ***H*** ^b^, ddd, ^2^ *J*_H6b,H6a_ = 12.9 Hz/^3^ *J*_H6b,NH_ = 4.8 Hz/^3^ *J*_H6b,H5_ = 1.5 Hz, 1H), 3.38–3.33 (C^3^ ***H*** + C^5^ ***H*** overlapped, m, 2H), 3.24 (OC ***H***_3_, s, 3H), 3.22–3.17 (C^2^ ***H*** + C^2′, 6′^ ***H***_ax_ overlapped, m, 3H), 3.05–2.98 (C^6^ ***H*** ^a^, m, 1H), 2.90 (C^4^ ***H***, ddd, ^3^ *J*_H4,H5_ = 9.2 Hz/^3^ *J*_H4,H3_ = 9.4 Hz/^3^ *J*_H4,OH4_ = 5.5 Hz, 1H), 2.57–2.50 (C^4′^ ***H***, m, 1H), 1.72 (C^3′, 5′^ ***H***_eq_, br m, 2H), 1.58 (C^3′, 5′^ ***H***_ax_, br q, ^2^ *J*_H3′/5′ax,H3′/5′eq_ = 13.4 Hz, 2H), 1.42 (N^1",3"^CH_2_C ***H***_3_, t, ^3^ *J*_H,H_ = 7.0 Hz, 6H) ppm. ^13^C{^1^H} NMR (100 MHz; DMSO‐D_6_, 298 K): *δ* 204.4 (N ***C*** SS), 184.0 (***C*** ^2"^),174.0 (***C*** =O), 132.4 (***C*** ^8",9"^), 124.2 (***C*** ^5",6"^H), 112.0 (***C*** ^4",7"^H), 99.7 (***C*** ^1^H), 73.0 (***C*** ^3^H), 72.1 (***C*** ^4^H), 72.0 (***C*** ^2^H), 70.3 (***C*** ^5^H), 54.3 (O ***C*** H_3_), 50.5 (***C*** ^2′, 6′^H_2_), 43.2 (N^1",3"^ ***C*** H_2_), 40.9 (***C*** ^4′^H), 39.8 (***C*** ^6^H_2_), 28.5 (***C*** ^3′, 5′^H_2_), 15.5 (N^1",3"^CH_2_ ***C*** H_3_) ppm. UV–Vis (DMF, 20 μM, 298 K): *λ*_max_ (log *ε*) 272 (4.30, π*←*π* intraligand/BzImy), 281_sh_ (4.43, π*←*π* intraligand/BzImy), 287 (4.54, π*←*π* intraligand/NCSS + π*←*π* intraligand/BzImy overlapped), 305 (4.06, *p*(Au)←*p*(S) LMCT) nm.^[^ 77, 78, 79, 80, 81, 82, 83–84 ^]^

## Supporting Information

Supporting Information available: materials, methods and instrumentation, synthesis and characterization of the zinc(II)‐dithiocarbamato intermediates **Zn1‐5**, the gold(I) precursors [AuICl(PPh3)] and [AuICl(Et2BzImy)], the gold(III) complexes **Au1‐5**, the gold(I)‐phosphine complexes **AuP4‐5** and the ligand (PPh4)(SSC‐Sar‐OEt), crystallographic data of (PPh4)(SSC‐Sar‐OEt), **AuP2**, **AuC1** and **AuC2**. The authors have cited additional references within the Supporting Information.^[^ ^77^ ^–^ ^84^ ^]^

## Conflict of Interest

The authors declare no conflict of interest.

## Supporting information

Supplementary Material

## Data Availability

The data that support the findings of this study are available in the *supplementary material* of this article.
