# Supplementary material for: Gold‐Dithiocarbamato Glycoconjugates as Potential Anticancer Agents: Design, Physico‐Chemical Characterization, and In Vitro Biological Activity
Source: Chembiochem. 2025 Aug 26;26(22):e202500447. doi: 10.1002/cbic.202500447 (PMC12631006; doi:10.1002/cbic.202500447)
Supplement: Supplementary file 1 — Supplementary Material [file CBIC-26-e202500447-s001.pdf]

Supporting Information

Gold-Dithiocarbamato Glycoconjugates as Potential Anticancer Agents: Design, Physico-Chemical Characterization and *In Vitro* Biological Activity

Andrea Pettenuzzo,[a] Jessica Wölker,[b] Luciano Marchiò,[c] Ingo Ott,*[b] and Luca Ronconi*[a]

[a] School of Biological and Chemical Sciences, University of Galway, University Road, Galway H91 TK33, Ireland

[b] Institute of Medicinal and Pharmaceutical Chemistry, Technische Universität Braunschweig, Beethovenstr. 55, 38106 Braunschweig, Germany

[c] Department of Chemistry, Life Sciences and Environmental Sustainability, University of Parma, Parco Area delle Scienze 11/a, 43124 Parma, Italy

Table of Contents

Materials and methods S2

Synthesis and characterization of the zinc(II)-dithiocarbamato intermediates **Zn1-5** S6

Synthesis and characterization of the gold(I) precursors [Au^ICl(PPh_3)] and [Au^ICl(Et_2BzImy)] S9

Synthesis and characterization of the gold(III) complexes **Au1-5** S10

Synthesis and characterization of the gold(I)-phosphine complexes **AuP4-5** S13

Synthesis, characterization and crystallographic data of the ligand (PPh_4)(SSC-Sar-OEt) (CCDC 2456096) S15

Crystallographic data of [Au^I(SSC-Inp-NH_2)(PPh_3)] (**AuP2**, CCDC 2456093), [Au^I(SSC-Sar-OEt)(Et_2BzImy)] (**AuC1**, CCDC 2456094) and [Au^I(SSC-Inp-NH_2)(Et_2BzImy)] (**AuC2**, CCDC 2456095) S17

Materials and methods

Abbreviations

Et_2BzImy: 1,3-diethylbenzimidazol-2-ylidene moiety

GlcN1: 2-NH-2-deoxy-D-glucose moiety

GlcN2: 1-*O*-methyl-2-NH-2-deoxy-D-glucopyranoside moiety

GlcN3: 1-*O*-methyl-6-NH-6-deoxy-D-glucopyranoside moiety

Inp: isonipecotic moiety

Sar: sarcosine (*N*-methylglycine) moiety

Materials

1-*O*-methyl-α-D-glucopyranoside (Acros), *N*-acetyl-(α,β)-D-glucosamine, *N*,*N*,*N′*,*N′*-tetramethyl-*O*-(*N*-succinimidyl)uronium tetrafluoroborate (Carbosynth), isonipecotamide, potassium tetrabromoaurate(III) dihydrate (Fisher), *N*-bromosuccinimide, chloro(dimethylsulfide)gold(I), chloro(triphenylphosphine)gold(I), *N*,*N*-diisopropylethylamine, zinc(II) acetate dihydrate (Fluorochem), amberlite IR120 (H^+ form) resin, benzimidazole, carbon disulfide, ethyl iodide, palladium on activated carbon ($10\%_w$), silver(I) oxide, tetraphenylphosphonium chloride (Sigma-Aldrich/Merck), (α,β)-D-glucosamine hydrochloride, ethyl isonipecotate, ethylsarcosine hydrochloride, isonipecotic acid (TCI), and all deuterated solvents for NMR analysis (Deutero) were of reagent grade or comparable purity and were used as supplied. Anhydrous DMF was obtained by passing the solvent over a column of alumina and subsequently stored over 4 Å activated molecular sieves under an inert atmosphere of nitrogen. All other reagents and solvents were used as purchased without any further purification. Phosphate buffered saline (PBS) solution (pH 7.4, 0.01 M phosphate buffer, 0.0027 M KCl, 0.137 M NaCl) was obtained by dissolving one tablet (Sigma-Aldrich) in 200 mL of Milli-Q water at room temperature.

Instrumentation

Thin layer chromatography (TLC) was performed on silica gel Merck $60F_{254}$ pre-coated aluminum sheets. Spots were visualized by direct UV irradiation at 254 nm or developed by exposure to either *p*-anisaldehyde or potassium permanganate staining solutions as appropriate.

Flash column chromatography was performed on Sigma Aldrich 60 Å silica gel (40-63 μm, 230-400 mesh) as stationary phase using the appropriate eluent.

Elemental analyses (carbon, hydrogen and nitrogen) were performed with a Perkin Elmer 2400 CHNS/O Series II analyzer.

Melting points were recorded on a Stuart SMP10 digital melting point apparatus and are uncorrected.

FT-IR spectra were recorded from CsI disks at room temperature on a Perkin Elmer Frontier FT-IR/FIR spectrophotometer in the range 4000-600 cm^{-1} (32 scans, resolution 4 cm^{-1}) and in the range 600-200 cm^{-1}

(32 scans, resolution 2 cm^{-1}). Data processing was carried out using OMNIC version 5.1 (Nicolet Instrument Corporation).

All ^{1}H, $^{13}C\{^{1}H\}$ and $^{31}P\{^{1}H\}$ NMR spectra were acquired in the appropriate deuterated solvent at room temperature on a Jeol ECX 400 MHz spectrometer. ^{1}H and ^{13}C signals were assigned with the aid of [^{1}H,^{1}H] COSY, $^{13}C\{^{1}H\}$ DEPT, [^{1}H,^{13}C] HMQC, [^{1}H,^{13}C] HSQC and [^{1}H,^{13}C] HMBC experiments where appropriate. ^{1}H and ^{13}C chemical shifts were referenced to TMS at 0.00 ppm *via* internal referencing to the residual peak of the deuterated solvent employed. ^{31}P chemical shifts were referenced to an external standard of 85% H_3PO_4 at 0 ppm. Data processing was carried out using MestReNova version 15.1 (Mestrelab Research S.L.).

UV-Vis spectra were acquired in the specified solvent at room temperature (unless otherwise stated) on a Thermo Scientific NanoDrop 2000C Spectrophotometer in the range 190-840 nm using a 1 cm pathlength quartz cuvette. Data processing was carried out using Spectragryph optical spectroscopy software version 1.2.[77]

Single crystal X-ray diffraction data were collected using a Bruker Smart instrument equipped with a Breeze area detector for [Au^{I}(SSC-Sar-OEt)(Et_2BzImy)] (**AuC1**), a Bruker D8 instrument equipped with a Photon II area detector for [Au^{I}(SSC-Inp-NH_2)(Et_2BzImy)] (**AuC2**) and [Au^{I}(SSC-Inp-NH_2)(PPh_3)] (**AuP2**), and a Rigaku Xcalibur instrument equipped with a sapphire detector for (PPh_4)(SSC-Sar-OEt) (Mo K_α radiation: λ = 0.71073 Å). Intensity data were integrated from several series of exposure frames and collected at different temperatures (200 K and 298 K) to cover the sphere of reciprocal lattice.[78] An absorption correction was applied using the software SADABS[79] for **AuC1**, **AuC2** and **AuP2**, and the software CrysAlisPro[80] for (PPh_4)(SSC-Sar-OEt). The crystal structures were solved with the ShelXT[81] structure solution program using intrinsic phasing, and refined with ShelXL on F^2 with full-matrix least squares, using the Olex2 software.[82] The peripheral ester group of the dithiocarbamato ligand in **AuC1** was found disordered in two positions, which were refined with site occupancy factors of 0.6 and 0.4, respectively. The asymmetric unit of **AuP2** comprises of two independent molecules connected through two hydrogen bonds involving the amido groups. Non-hydrogen atoms were refined with anisotropic thermal parameters and the hydrogen atoms were placed in their calculated positions. Graphical material was prepared with the Mercury 2021 software.[83] CCDC 2456093 (**AuP2**), CCDC 2456094 (**AuC1**), CCDC 2456095 (**AuC2**) and CCDC 2456096 ((PPh_4)(SSC-Sar-OEt)) contain the supplementary crystallographic data for this paper.

Cell lines and culture conditions

Human colorectal adenocarcinoma (HT-29; Cell Line Service, Eppelheim, Germany), metastatic breast adenocarcinoma (MDA-MB-231; Helmholtz Institute for Infection Research, Braunschweig, Germany) and breast adenocarcinoma (MCF-7; Cell Line Service, Eppelheim, Germany) cells were maintained in

high-glucose Dulbecco's Modified Eagle's Medium (4.5 g L^{-1} D-glucose, 4 mM L-glutamine, 1 mM sodium pyruvate, 1.5 g L^{-1} sodium bicarbonate; Life Technologies).

Human non-malignant kidney epithelial (RC-124) cells were maintained in McCoy's 5A Medium (modified to contain 1.5 mM L-glutamine and 2.2 g L^{-1} sodium bicarbonate; Life Technologies).

Cells were cultured at 37°C in 5% CO_2 and moisture-enriched atmosphere in the respective medium supplemented with 50 mg L^{-1} gentamicin and 10% v/v standardized fetal bovine serum superior (Biochrom) and passaged once a week.

For experiments involving RC-124 cells, microtiter plates were pre-treated as follows. 30 μL of a 1.5% m/v sterilized gelatine solution were added to each well of flat-bottomed 96-well plates, plates were covered with their lids and incubated for 1 h at 37°C. The excess solution was then removed, wells were washed with PBS solution (pH 7.4), and new cell culture medium was added.

Cell growth inhibition assay

Stock solutions of the gold(III) complexes **Au1-5**, and the gold(I) complexes **AuP1-5** and **AuC1-5** in DMF were freshly prepared and subsequently diluted with the appropriate complete cell culture medium to graded concentrations in such a way that the final amount of organic solvent did not exceed 0.1% v/v.

The *in vitro* antiproliferative activity of the various gold complexes was determined following a modified protocol previously reported.[68] 100 μL of HT-29 cells (2.5×10^3 cells mL^{-1}), MDA-MB-231 cells (4.0×10^3 cells mL^{-1}), MCF-7 cells (4.8×10^3 cells mL^{-1}), or RC-124 cells (1.4×10^3 cells mL^{-1}) were seeded in 96-well cell culture plates and incubated at 37°C in 5% CO_2 humidified atmosphere (note: for RC-124, pre-treated plates were used as mentioned above). After 72 h (MDA-MB-231, MCF-7, RC-124) or 48 h (HT-29), cells were treated with the test compounds at different concentrations (0.01-50 μM) and incubated for further 72 h (HT-29) or 96 h (MDA-MB-231, MCF-7, RC-124) at 37°C. The medium was then discarded, cells were washed with fresh PBS solution and harvested, and the Crystal Violet (Sigma-Aldrich/Merck) staining assay was performed to determine cell viability. The inhibition of cell growth induced by the tested compounds was assessed by measuring the absorbance of each well at 590 nm using a Victor X4 microplate reader (Perkin Elmer). Antiproliferative data are expressed as IC_{50} values, that is, the concentration (μM) of the test agent inhibiting cell proliferation by 50% compared with untreated control cultures. Results were calculated as the mean values ± standard deviation (SD) of three independent experiments.

Cellular uptake studies

Stock solutions of the of the gold(III) complexes **Au2** and **Au4**, and the gold(I) complexes **AuC2** and **AuC4** in DMF were freshly prepared and subsequently diluted with the appropriate complete cell culture medium to a concentration of 10 μM (along with 50 μM of GLUT1 inhibitor Cytochalasin B (Sigma-

Aldrich/Merck) where indicated) immediately before use in such a way that the final amount of organic solvent did not exceed 0.1% v/v.

The cellular uptake of selected gold complexes was determined following a protocol previously reported.[68,84] Two different cell culture media were used: high-glucose Dulbecco's Modified Eagle's Medium (4.5 g L^{-1} D-glucose, 4 mM L-glutamine, 1 mM sodium pyruvate, 1.5 g L^{-1} sodium bicarbonate; Life Technologies) and glucose-free Dulbecco's Modified Eagle's Medium (4 mM L-glutamine, 3.7 g L^{-1} sodium bicarbonate; Life Technologies). Both cell media were supplemented with 50 mg L^{-1} gentamicin and 10% v/v standardized fetal bovine serum superior (Biochrom).

MCF-7 cells were grown in 175 cm^2 cell culture flasks until at least 75-80% confluency. The cell culture medium was then replaced with 10 mL of fresh medium containing the test compound (and Cytochalasin B where indicated), and the flasks were incubated at 37°C in 5% CO_2 humidified atmosphere for 6 h. After the incubation period, the uptake was stopped by removing the cell culture medium. Cells were then washed with PBS (10 mL), the washing solution removed, and cells were isolated with a scratcher, resuspended in PBS (10 mL) and centrifuged (5 min at 1096g). The cell pellets were stored at -20°C for further use. For metal quantification, pellets were resuspended in demineralized water (200 μL) and lysed for 30 min by ultrasonication.

Atomic absorption spectroscopy (AAS) measurements

For the measurement of gold contents a contrAA 700 high-resolution continuum-source atomic absorption spectrometer (Analytik Jena) was used. Pure samples of the respective gold complexes were used as standards and calibration was done in a matrix-matched manner (meaning that all samples and standards were adjusted to the same protein concentration by dilution with distilled water).

Triton-X 100 (1%, 10 μL) and ascorbic acid (1%, 10 μL), were added directly to each sample (100 μL). Samples (25 μL) were injected into coated standard graphite tubes (Analytik Jena) and thermally processed as previously described.[68] Drying steps were adjusted and the atomization temperature set to 1850°C. Gold was quantified at a wavelength of 242.79 nm. The mean integrated absorbances of triplicate injections were used throughout the studies. The final gold contents were calculated from the data obtained in two independent experiments and are expressed as nmol of Au per mg of cellular protein.

Synthesis and characterization of the zinc(II) intermediates Zn1-5

All zinc(II)-dithiocarbamato intermediates were synthesized as previously reported in the literature.[38]

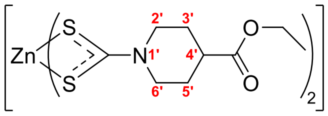

Zn1

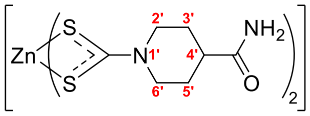

Zn2

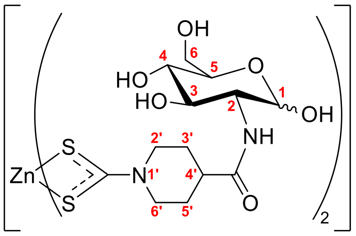

Zn3

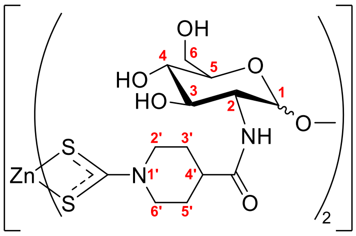

Zn4

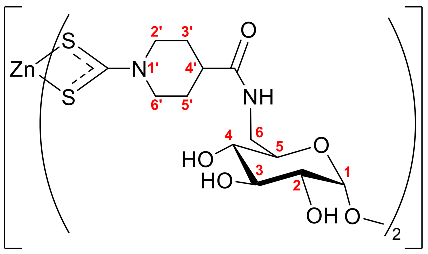

Zn5

[Zn^{II}(SSC-Inp-OEt)$_2$] (Zn1). FT-IR (CsI disk, 298 K): $\tilde{\nu}_{max}$ 1733 (ν, C=O), 1494 (ν, N–CSS), 1176 (ν, C–OEt), 1042 (ν, O–Et), 1007 (ν_a, SCS), 568 (ν_s, SCS), 398 (ν_a, ZnS$_4$) cm^{-1}. ^{1}H NMR (400 MHz, DMSO-D$_6$, 298 K): δ 4.72 (C$^{2',6'}$***H***$_{eq}$, dt, $^2J_{H2'/6'eq,H2'/6'ax}$ = 13.0 Hz/$^3J_{H2'/6'eq,H3'/5'}$ = 3.3 Hz, 4H), 4.08 (OC***H***$_2$, q, $^3J_{H,H}$ = 7.1 Hz, 4H), 3.39 (C$^{2',6'}$***H***$_{ax}$, td, $^2J_{H2'/6'ax,H2'/6'eq}$ = 12.9 Hz/$^3J_{H2'/6'ax,H3'/5'}$ = 2.4 Hz, 4H), 2.68 (C$^{4'}$***H***, tt, $^3J_{H4',H3'/5'ax}$ = 10.9 Hz/$^3J_{H4',H3'/5'eq}$ = 4.1 Hz, 2H), 1.94 (C$^{3',5'}$***H***$_{eq}$, dd, $^2J_{H3'/5'eq,H3'/5'ax}$ = 13.4 Hz/$^3J_{H3'/5'eq,H2'/6'}$ = 3.0 Hz, 4H), 1.57 (C$^{3',5'}$***H***$_{ax}$, qd, $^2J_{H3'/5'ax,H3'/5'eq}$ = 13.2 Hz/$^3J_{H3'/5'ax,H2'/6'}$ = 3.8 Hz, 4H), 1.19 (C***H***$_3$, t, $^3J_{H,H}$ = 7.1 Hz, 6H) ppm. ^{13}C{^{1}H} NMR (100 MHz; DMSO-D$_6$, 298 K): δ 202.6 (N***C***SS), 173.6 (***C***=O), 60.2 (O***C***H$_2$), 50.4 (***C***$^{2',6'}$H$_2$), 38.9 (***C***$^{4'}$H), 27.8 (***C***$^{3',5'}$H$_2$), 14.1 (***C***H$_3$) ppm.

[Zn^{II}(SSC-Inp-NH$_2$)$_2$] (Zn2). FT-IR (CsI disk, 298 K): $\tilde{\nu}_{max}$ 3439/3208 ($\nu_{a,s}$, NH$_2$), 1667 (ν, C=O (amide I)), 1613 (δ_{ip}, CNH$_2$ (amide II)), 1492 (ν, N–CSS), 1007 (ν_a, SCS), 561 (ν_s, SCS), 392 (ν_a, ZnS$_4$) cm^{-1}. ^{1}H NMR (400 MHz, DMSO-D$_6$, 298 K): δ 7.36 (N***H***$_{cis}$, s, 2H), 6.87 (N***H***$_{trans}$, s, 2H), 4.82 (C$^{2',6'}$***H***$_{eq}$, br d, $^2J_{H2'/6'eq,H2'/6'ax}$ = 12.9 Hz, 4H), 3.26 (C$^{2',6'}$***H***$_{ax}$, td, $^2J_{H2'/6'ax,H2'/6'eq}$ = 11.4 Hz/$^3J_{H2'/6'ax,H3'/5'}$ = 1.6 Hz, 4H), 2.40 (C$^{4'}$***H***, tt, $^3J_{H4',H3'/5'ax}$ = 11.2 Hz/$^3J_{H4',H3'/5'eq}$ = 3.9 Hz, 2H), 1.82 (C$^{3',5'}$***H***$_{eq}$, dd, $^2J_{H3'/5'eq,H3'/5'ax}$ = 14.2 Hz/$^3J_{H3'/5'eq,H2'/6'}$ = 2.9 Hz, 4H), 1.54 (C$^{3',5'}$***H***$_{ax}$, qd, $^2J_{H3'/5'ax,H3'/5'eq}$ = 12.4 Hz/$^3J_{H3'/5'ax,H2'/6'}$ = 3.1 Hz, 4H) ppm. ^{13}C{^{1}H} NMR (100 MHz; DMSO-D$_6$, 298 K): δ 202.3 (N***C***SS), 175.6 (***C***=O), 50.7 (***C***$^{2',6'}$H$_2$), 40.1 (***C***$^{4'}$H), 28.4 (***C***$^{3',5'}$H$_2$) ppm.

[Zn^{II}(SSC-Inp-GlcN1)$_2$] (Zn3). FT-IR (CsI disk, 298 K): $\tilde{\nu}_{max}$ 3293 (ν, OH + NH overlapped), 1638 (ν, C=O (amide I)), 1547 (δ_{ip}, CNH (amide II)), 1487 (ν, N–CSS), 1071/1060 (ν, C–OH), 1010 (ν_a, SCS), 570 (ν_s, SCS), 380 (ν_a, ZnS$_4$) cm^{-1}. ^{1}H NMR (400 MHz, DMSO-D$_6$, 298 K): δ 7.70 (N***H*** β, d, $^3J_{NH,H2}$ = 8.7 Hz, 0.3H), 7.66 (N***H*** α, d, $^3J_{NH,H2}$ = 7.9 Hz, 2H), 6.49 (C^{1}O***H*** β, d, $^3J_{OH1,H1}$ = 6.4 Hz, 0.3H), 6.42 (C^{1}O***H*** α, d, $^3J_{OH1,H1}$ = 4.1 Hz, 2H), 4.94-4.90 (C^{3}O***H*** α + C^1***H*** α + C^{4}O***H*** β overlapped, m, 4.3H), 4.85-

4.78 (C$^{2',6'}$***H***$_{eq}$ α and β + C^{3}O***H*** β overlapped, m, 4.6H), 4.61 (C^{4}O***H*** α, d, $^{3}J_{OH4,H4}$ = 5.6 Hz, 2H), 4.53 (C^{6}O***H*** β, t, $^{3}J_{OH6,H6(dia)}$ = 5.9 Hz, 0.3H), 4.46 (C^{1}***H*** β, d, $^{3}J_{H1,H2}$ = 6.7 Hz, 0.3H), 4.43 (C^{6}O***H*** α, t, $^{3}J_{OH6,H6(dia)}$ = 5.8 Hz, 2H), 3.69-3.38 (C^{2}***H*** α + C^{4}***H*** α and β + C^{5}***H*** α + C^{6}***H***$_{2}$ α and β overlapped, m, 10.9H), 3.31-3.22 (C$^{2',6'}$***H***$_{ax}$ α and β + C^{2}***H*** β + C^{3}***H*** β overlapped, m, 5.2H), 3.17-3.03 (C^{3}***H*** α + C^{5}***H*** β overlapped, m, 2.3H), 2.56-2.53 (C$^{4'}$***H*** α and β overlapped, m, 2.3H), 1.78 (C$^{3',5'}$***H***$_{eq}$ α and β overlapped, br t, 4.6H), 1.57 (C$^{3',5'}$***H***$_{ax}$ α and β overlapped, br q, 4.6H) ppm. ^{13}C{^{1}H} NMR (100 MHz; DMSO-D$_{6}$, 298 K): δ 202.4 (N***C***SS), 173.8 (***C***=O), 95.4 (***C***^{1}H β), 90.5 (***C***^{1}H α), 76.9 (***C***^{5}H β), 74.2 (***C***^{3}H β), 72.1 (***C***^{5}H α), 71.1 (***C***^{3}H α), 70.9 (***C***^{4}H β), 70.5 (***C***^{4}H α), 61.3 (***C***^{6}H$_{2}$ β), 61.1 (***C***^{6}H$_{2}$ α), 57.0 (***C***^{2}H β), 54.3 (***C***^{2}H α), 50.7 (***C***$^{2',6'}$H$_{2}$), 40.0 (***C***$^{4'}$H), 28.5 (***C***$^{3',5'}$H$_{2}$) ppm. No differentiation of the ^{13}C signals of the isonipecotic moiety due to the presence of both α and β anomers could be observed. Solution α:β anomers ratio ≈6.7:1 (based on the ^{1}H NMR spectrum).

[Zn^{II}(SSC-Inp-GlcN2)$_{2}$] (Zn4). FT-IR (CsI disk, 298 K): $\tilde{\nu}_{max}$ 3319 (ν, OH + NH overlapped), 1645 (ν, C=O (amide I)), 1557 (δ_{ip}, CNH (amide II)), 1494 (ν, N–CSS), 1062/1040 (ν, C–OH + C^{1}–O–CH$_{3}$ overlapped), 1007 (ν_{a}, SCS), 576 (ν_{s}, SCS), 382 (ν_{a}, ZnS$_{4}$) cm^{-1}. ^{1}H NMR (400 MHz, DMSO-D$_{6}$, 298 K): δ 7.80 (N***H***, d, $^{3}J_{NH,H2}$ = 8.1 Hz, 2H), 5.01 (C^{4}O***H***, d, $^{3}J_{OH4,H4}$ = 5.6 Hz, 2H), 4.82 (C$^{2',6'}$***H***$_{eq}$, br d, $^{2}J_{H2'/6'eq,H2'/6'ax}$ = 12.7 Hz, 4H), 4.73 (C^{3}O***H***, d, $^{3}J_{OH3,H3}$ = 5.9 Hz, 2H), 4.55 (C^{6}O***H***, t, $^{3}J_{OH6,H6(dia)}$ = 6.0 Hz, 2H), 4.53 (C^{1}***H***, d, $^{3}J_{H1,H2}$ = 3.3 Hz, 2H), 3.67-3.42 (C^{2}***H*** + C^{3}***H*** + C^{6}***H***$_{2}$ overlapped, m, 8H), 3.33-3.24 (C^{5}***H*** + C$^{2',6'}$***H***$_{ax}$ overlapped, m, 6H), 3.24 (OC***H***$_{3}$, s, 6H), 3.12 (C^{4}***H***, ddd, $^{3}J_{H4,H5}$ = 10.0 Hz/$^{3}J_{H4,H3}$ = 9.4 Hz/$^{3}J_{H3,OH3}$ = 5.6 Hz, 2H), 2.55 (C$^{4'}$***H***, tt, $^{3}J_{H4',H3'/5'ax}$ = 11.0 Hz/$^{3}J_{H4',H3'/5'eq}$ = 3.5 Hz, 2H), 1.78 (C$^{3',5'}$***H***$_{eq}$, br t, $^{2}J_{H3'/5'eq,H3'/5'ax}$ = 10.9 Hz, 4H), 1.58 (C$^{3',5'}$***H***$_{ax}$, br qd, $^{2}J_{H3'/5'ax,H3'/5'eq}$ = 10.1 Hz/$^{3}J_{H3'/5'ax,H2'/6'}$ = 3.4 Hz, 4H) ppm. The ^{1}H NMR signals refer to the α anomer only. Signals related to the β anomer were hardly detectable; only a very few were observed and could be undoubtedly assigned (such as δ(N***H***) = 7.68, δ(C^{3}O***H***) = 4.91, δ(C^{1}***H***) = 4.19 ($^{3}J_{H1,H2}$ = 8.4 Hz), and δ(OC***H***$_{3}$) = 3.58 ppm. ^{13}C{^{1}H} NMR (100 MHz; DMSO-D$_{6}$, 298 K): δ 202.2 (N***C***SS), 173.9 (***C***=O), 97.9 (***C***^{1}H), 72.8 (***C***^{5}H), 70.8 (***C***^{3}H), 70.7 (***C***^{4}H), 60.9 (***C***^{6}H$_{2}$), 54.5 (O***C***H$_{3}$), 53.8 (***C***^{2}H), 50.8 (***C***$^{2',6'}$H$_{2}$), 40.0 (***C***$^{4'}$H), 28.5 (***C***$^{3',5'}$H$_{2}$) ppm. No ^{13}C signals assignable to the β anomer were detected. Solution α:β anomers ratio ≈25:1 (based on the ^{1}H NMR spectrum).

[Zn^{II}(SSC-Inp-GlcN3)$_{2}$] (Zn5). FT-IR (CsI disk, 298 K): $\tilde{\nu}_{max}$ 3392 (ν, OH + NH overlapped), 1637 (ν, C=O (amide I)), 1543 (δ_{ip}, CNH (amide II)), 1494 (ν, N–CSS), 1077/1050 (ν, C–OH + C^{1}–O–CH$_{3}$ overlapped), 1010 (ν_{a}, SCS), 564 (ν_{s}, SCS), 366 (ν_{a}, ZnS$_{4}$) cm^{-1}. ^{1}H NMR (400 MHz, DMSO-D$_{6}$, 298 K): δ 7.97 (N***H***, t, $^{3}J_{NH,H6(dia)}$ = 5.8 Hz, 2H), 4.97 (C^{4}O***H***, d, $^{3}J_{OH4,H4}$ = 5.5 Hz, 2H), 4.84 (C$^{2',6'}$***H***$_{eq}$, br m, 4H), 4.81 (C^{3}O***H***, d, $^{3}J_{OH3,H3}$ = 4.8 Hz, 2H), 4.75 (C^{2}O***H***, d, $^{3}J_{OH2,H2}$ = 6.5 Hz, 2H), 4.51 (C^{1}***H***, d, $^{3}J_{H1,H2}$ = 3.6 Hz, 2H), 3.55 (C^{6}***H***^{b}, ddd, $^{2}J_{H6b,H6a}$ = 13.6 Hz/$^{3}J_{H6b,NH}$ = 5.8 Hz/$^{3}J_{H6b,H5}$ = 2.0 Hz, 2H), 3.40-3.35 (C^{3}***H*** +

C^5***H*** overlapped, m, 4H), 3.24 (OC$\boldsymbol{H}_3$, s, 6H), 3.28-3.19 (C^2***H*** + $C^{2',6'}\boldsymbol{H}_{ax}$ overlapped, m, 6H), 3.01 ($C^6\boldsymbol{H}^a$, ddd, $^2J_{H6b,H6a}$ = 13.3 Hz/$^3J_{H6b,NH}$ = 5.9 Hz/$^3J_{H6b,H5}$ = 1.6 Hz, 2H), 2.90 (C^4***H***, ddd, $^3J_{H4,H5}$ = 10.1 Hz/$^3J_{H4,H3}$ = 9.4 Hz/$^3J_{H4,OH4}$ = 5.4 Hz, 2H), 2.57-2.52 ($C^{4'}$***H***, m, 2H), 1.78-1.75 ($C^{3',5'}\boldsymbol{H}_{eq}$, br m, 4H), 1.58 ($C^{3',5'}\boldsymbol{H}_{ax}$, br qd, $^2J_{H3'/5'ax,H3'/5'eq}$ = 11.8 Hz/$^3J_{H3'/5'ax,H2'/6'}$ = 3.7 Hz, 4H) ppm. $^{13}C\{^1H\}$ NMR (100 MHz; DMSO-D_6, 298 K): δ 202.2 (N***C***SS), 173.8 (***C***=O), 99.6 ($\boldsymbol{C}^1$H), 73.0 ($\boldsymbol{C}^3$H), 72.1 ($\boldsymbol{C}^4$H), 72.0 ($\boldsymbol{C}^2$H), 70.3 ($\boldsymbol{C}^5$H), 54.3 (O***C***H_3), 50.7 ($\boldsymbol{C}^{2',6'}H_2$), 39.9 ($\boldsymbol{C}^{4'}$H), 39.8 ($\boldsymbol{C}^6H_2$), 28.4 ($\boldsymbol{C}^{3',5'}H_2$) ppm.

Synthesis and characterization of the gold(I) precursors [$Au^{I}Cl(PPh_3)$] and [$Au^{I}Cl(Et_2BzImy)$]

Chloro(triphenylphosphine)gold(I) ([$Au^{I}Cl(PPh_3)$])

Commercially available (98%).

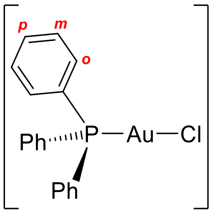

FT-IR (CsI disk, 298 K): $\tilde{\nu}_{max}$ 1102 ($\nu_{q\ vib}$, P–Ph_3), 713/693 ($\nu_{r\ vib}$, P–Ph_3), 547/501 ($\delta_{y\ vib}$, P–Ph_3), 449 ($\nu_{t\ vib}$, P–Ph_3), 399 (ν, Au–P), 330/323 (ν, Au–$^{35/37}$Cl), 276/242 ($\delta_{x\ vib}$, P–Ph_3) cm^{-1}.[43,44] ^{1}H NMR (400 MHz, $CDCl_3$, 298 K): δ 7.56-7.45 (C***H*** Ph, m, 15H) ppm.[38] ^{13}C{^{1}H} NMR (100 MHz; $CDCl_3$, 298 K): δ 134.3 (*o*-***C***H, d, $^{2}J_{C,P}$ = 14.6 Hz), 132.1 (*p*-***C***H, d, $^{4}J_{C,P}$ = 2.5 Hz), 129.4 (*m*-***C***H, d, $^{3}J_{C,P}$ = 12.0 Hz), 128.9 (***C***P, d, $^{1}J_{C,P}$ = 62.3 Hz) ppm.[38] ^{31}P{^{1}H} NMR (162 MHz; $CDCl_3$, 298 K): δ 33.8 (Au***P***Ph_3) ppm.[38]

Chloro(1,3-diethyl-1,3-dihydro-2*H*-benzimidazol-2-ylidene)gold(I) ([$Au^{I}Cl(Et_2BzImy)$])

Synthesized as previously reported in the literature.[69]

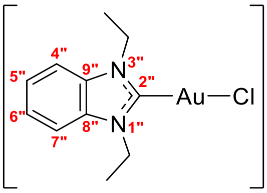

FT-IR (CsI disk, 298 K): $\tilde{\nu}_{max}$ 1461 (ν, C=N), 1086 (ν, C–N), 1044 (ν, N–Et), 566 (ν, C–Au), 340/333(ν, Au–$^{35/37}$Cl) cm^{-}.[43,48,49,50,52,53] ^{1}H NMR (400 MHz, $CDCl_3$, 298 K): δ 7.51-7.41 ($C^{4'',7''}$***H*** + $C^{5'',6''}$***H*** overlapped, m, 4H), 4.54 ($N^{1'',3''}$C***H***$_2$, q, $^{3}J_{H,H}$ = 7.3 Hz, 4H), 1.54 ($N^{1'',3''}CH_2$C***H***$_3$, t, $^{3}J_{H,H}$ = 7.0 Hz, 6H) ppm.[69] ^{13}C{^{1}H} NMR (100 MHz; $CDCl_3$, 298 K): δ 177.8 (***C***$^{2''}$), 132.9 (***C***$^{8'',9''}$), 124.6 (***C***$^{5'',6''}$H), 111.5 (***C***$^{4'',7''}$H), 44.1 ($N^{1'',3''}$***C***H_2), 15.6 ($N^{1'',3''}CH_2$***C***H_3) ppm.[69]

Synthesis and characterization of the gold(III) complexes Au1-5

All gold(III)-dithiocarbamato complexes were synthesized as previously reported in the literature.[38]

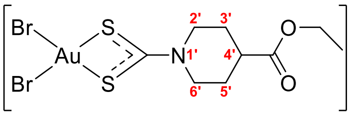

Au1

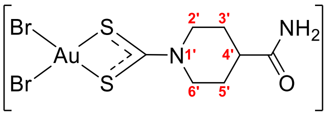

Au2

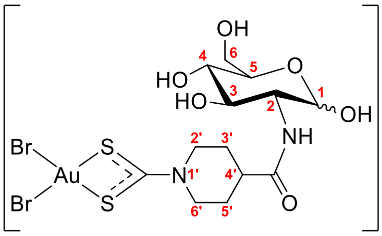

Au3

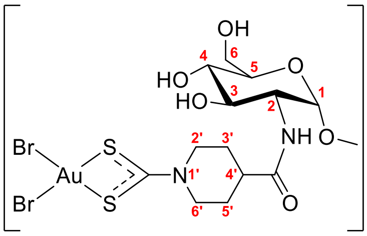

Au4

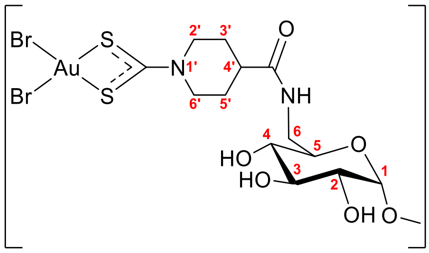

Au5

[Au^{III}Br$_2$(SSC-Inp-OEt)] (Au1). FT-IR (CsI disk, 298 K): $\tilde{\nu}_{max}$ 1729 (ν, C=O), 1571 (ν, N–CSS), 1183 (ν, C–OEt), 1041 (ν, O–Et), 1005 (ν_a, SCS), 536 (ν_s, SCS), 375 (ν_a, SAuS), 351 (ν_s, SAuS), 253 (ν_a, BrAuBr), 228 (ν_s, BrAuBr) cm^{-1}. ^{1}H NMR (400 MHz, DMSO-D$_6$, 298 K): δ 4.09 (OC$\boldsymbol{H}_2$, q, $^3J_{H,H}$ = 7.1 Hz, 2H), 4.06 (C$^{2',6'}\boldsymbol{H}_{eq}$, dt, $^2J_{H2'/6'eq,H2'/6'ax}$ = 6.8 Hz/$^3J_{H2'/6'eq,H3'/5'}$ = 3.7 Hz, 2H), 3.61 (C$^{2',6'}\boldsymbol{H}_{ax}$, td, $^2J_{H2'/6'ax,H2'/6'eq}$ = 12.9 Hz/$^3J_{H2'/6'ax,H3'/5'}$ = 3.3 Hz, 2H), 2.90 (C$^{4'}\boldsymbol{H}$, tt, $^3J_{H4',H3'/5'ax}$ = 12.4 Hz/$^3J_{H4',H3'/5'eq}$ = 4.0 Hz, 1H), 2.08 (C$^{3',5'}\boldsymbol{H}_{eq}$, dd, $^2J_{H3'/5'eq,H3'/5'ax}$ = 13.9 Hz/$^3J_{H3'/5'eq,H2'/6'}$ = 3.6 Hz, 2H), 1.75 (C$^{3',5'}\boldsymbol{H}_{ax}$, qd, $^2J_{H3'/5'ax,H3'/5'eq}$ = 11.1 Hz/$^3J_{H3'/5'ax,H2'/6'}$ = 4.1 Hz, 2H), 1.19 (C$\boldsymbol{H}_3$, t, $^3J_{H,H}$ = 7.1 Hz, 3H) ppm. ^{13}C{^{1}H} NMR (100 MHz; DMSO-D$_6$, 298 K): δ 187.3 (N$\boldsymbol{C}$SS), 172.6 ($\boldsymbol{C}$=O), 60.4 (O$\boldsymbol{C}$H$_2$), 48.1 ($\boldsymbol{C}^{2',6'}$H$_2$), 39.0 ($\boldsymbol{C}^{4'}$H), 26.8 ($\boldsymbol{C}^{3',5'}$H$_2$), 14.1 ($\boldsymbol{C}$H$_3$) ppm. UV-Vis (DMSO, 50 μM, 298 K): λ_{max} (log ε) 273 (4.41, π*←π intraligand/NCSS), 283 (4.44, π*←π intraligand/NCSS), 371 (3.60, d←d Au(III)) nm.[47,57,58]

[Au^{III}Br$_2$(SSC-Inp-NH$_2$)] (Au2). FT-IR (CsI disk, 298 K): $\tilde{\nu}_{max}$ 3408/3176 ($\nu_{a,s}$, NH$_2$), 1660 (ν, C=O (amide I)), 1620 (δ_{ip}, CNH$_2$ (amide II)), 1565 (ν, N–CSS), 1007 (ν_a, SCS), 535 (ν_s, SCS), 376 (ν_a, SAuS), 351 (ν_s, SAuS), 250 (ν_a, BrAuBr), 230 (ν_s, BrAuBr) cm^{-1}. ^{1}H NMR (400 MHz, DMSO-D$_6$, 298 K): δ 7.40 (N$\boldsymbol{H}_{cis}$, s, 1H), 6.97 (N$\boldsymbol{H}_{trans}$, s, 1H), 4.12 (C$^{2',6'}\boldsymbol{H}_{eq}$, dt, $^2J_{H2'/6'eq,H2'/6'ax}$ = 13.5 Hz/$^3J_{H2'/6'eq,H3'/5'}$ = 3.1 Hz, 2H), 3.57 (C$^{2',6'}\boldsymbol{H}_{ax}$, td, $^2J_{H2'/6'ax,H2'/6'eq}$ = 12.5 Hz/$^3J_{H2'/6'ax,H3'/5'}$ = 3.1 Hz, 2H), 2.62 (C$^{4'}\boldsymbol{H}$, tt, $^3J_{H4',H3'/5'ax}$ = 10.5 Hz/$^3J_{H4',H3'/5'eq}$ = 3.7 Hz, 1H), 1.97 (C$^{3',5'}\boldsymbol{H}_{eq}$, dd, $^2J_{H3'/5'eq,H3'/5'ax}$ = 13.8 Hz/$^3J_{H3'/5'eq,H2'/6'}$ = 3.3 Hz, 2H), 1.69 (C$^{3',5'}\boldsymbol{H}_{ax}$, qd, $^2J_{H3'/5'ax,H3'/5'eq}$ = 11.6 Hz/$^3J_{H3'/5'ax,H2'/6'}$ = 4.1 Hz, 2H) ppm. ^{13}C{^{1}H} NMR (100 MHz; DMSO-D$_6$, 298 K): δ 186.9 (N$\boldsymbol{C}$SS), 174.5 ($\boldsymbol{C}$=O), 48.4 ($\boldsymbol{C}^{2',6'}$H$_2$), 40.3 ($\boldsymbol{C}^{4'}$H), 27.5 ($\boldsymbol{C}^{3',5'}$H$_2$) ppm. UV-Vis (DMSO, 50 μM, 298 K): λ_{max} (log ε) 272 (4.16, π*←π intraligand/NCSS), 281 (4.18, π*←π intraligand/NCSS), 321$_{sh}$ (3.67, π*←d MLCT), 362 (3.42, d←d Au(III)) nm.

[Au^{III}Br$_2$(SSC-Inp-GlcN1)] (Au3). FT-IR (CsI disk, 298 K): $\tilde{\nu}_{max}$ 3306 (ν, OH + NH overlapped), 1636 (ν, C=O (amide I)), 1561 (ν, N–CSS), 1536 (δ_{ip}, CNH (amide II)), 1059 (ν, C–OH), 1007 (ν_a, SCS), 533 (ν_s, SCS), 392 (ν_a, SAuS), 357 (ν_s, SAuS), 249 (ν_a, BrAuBr), 227 (ν_s, BrAuBr) cm^{-1}. ^{1}H NMR (400 MHz, DMSO-D$_6$, 298 K): δ 7.75 (N***H*** β, d, $^3J_{NH,H2}$ = 9.4 Hz, 0.2H), 7.73 (N***H*** α, d, $^3J_{NH,H2}$ = 8.2 Hz, 1H), 6.45 (C^{1}O***H*** α and β overlapped, br s, 1.2H), 4.91 (C^1***H*** α, d, $^3J_{H1,H2}$ = 2.8 Hz, H), 4.45 (C^1***H*** β, d, $^3J_{H1,H2}$ = 8.1 Hz, 0.2H), 4.13 (C$^{2',6'}$***H***$_{eq}$ α and β overlapped, br dt, $^2J_{H2'/6'eq,H2'/6'ax}$ = 13.3 Hz/$^3J_{H2'/6'eq,H3'/5'}$ = 4.3 Hz, 2.4H), ~4.0 (C^{3}O***H*** + C^{4}O***H*** + C^{6}O***H*** α and β overlapped, vbr s, 3.6H), 3.69-3.43 (C^2***H*** α + C^4***H*** α and β + C^5***H*** α + C^6***H***$_2$ α and β + C$^{2',6'}$***H***$_{ax}$ α and β overlapped, m, 8H), 3.42-3.24 (C^2***H*** β + C^3***H*** β overlapped, m, 0.4H), 3.16-3.04 (C^3***H*** α + C^5***H*** β overlapped, m, 1.2H), 2.75 (C$^{4'}$***H*** α, tt, $^3J_{H4',H3'/5'ax}$ = 10.2 Hz/$^3J_{H4',H3'/5'eq}$ = 3.7 Hz, 1H), 2.63 (C$^{4'}$***H*** β, tt, $^3J_{H4',H3'/5'ax}$ = 9.9 Hz/$^3J_{H4',H3'/5'eq}$ = 3.8 Hz, 0.2H), 1.95 (C$^{3',5'}$***H***$_{eq}$ α and β overlapped, td, $^2J_{H3'/5'eq,H3'/5'ax}$ = 14.3 Hz/$^3J_{H3'/5'eq,H2'/6'}$ = 2.9 Hz, 2.4H), 1.71 (C$^{3',5'}$***H***$_{ax}$ α and β overlapped, qd, $^2J_{H3'/5'ax,H3'/5'eq}$ = 10.0 Hz/$^3J_{H3'/5'ax,H2'/6'}$ = 3.1 Hz, 2.4H) ppm. ^{13}C{^{1}H} NMR (100 MHz; DMSO-D$_6$, 298 K): δ 186.9 (N***C***SS α; β not detected), 172.7 (***C***=O α), 172.6 (***C***=O β), 95.3 (***C***^{1}H β), 90.5 (***C***^{1}H α), 76.9 (***C***^{5}H β), 74.2 (***C***^{3}H β), 72.1 (***C***^{5}H α), 71.1 (***C***^{3}H α), 70.8 (***C***^{4}H β), 70.4 (***C***^{4}H α), 61.2 (***C***^{6}H$_2$ β), 61.1 (***C***^{6}H$_2$ α), 57.0 (***C***^{2}H β), 54.3 (***C***^{2}H α), 48.4 (***C***$^{2',6'}$H$_2$ α), 48.3 (***C***$^{2',6'}$H$_2$ β), 40.7 (***C***$^{4'}$H α), 40.4 (***C***$^{4'}$H β), 27.7 (***C***$^{3',5'}$H$_2$ α), 27.6 (***C***$^{3',5'}$H$_2$ β) ppm. Solution α:β anomers ratio ≈5:1 (based on the ^{1}H NMR spectrum). UV-Vis (DMSO, 50 μM, 298 K): λ_{max} (log ε) 272 (4.28, π*←π intraligand/NCSS), 277 (4.30, π*←π intraligand/NCSS), 319$_{sh}$ (3.91, π*←d MLCT), 368 (3.40, d←d Au(III)) nm.

[Au^{III}Br$_2$(SSC-Inp-GlcN2)] (Au4). FT-IR (CsI disk, 298 K): $\tilde{\nu}_{max}$ 3364 (ν, OH + NH overlapped), 1649 (ν, C=O (amide I)), 1561 (ν, N–CSS), 1533 (δ_{ip}, CNH (amide II)), 1059/1040 (ν, C–OH + C^1–O–CH$_3$ overlapped), 1008 (ν_a, SCS), 532 (ν_s, SCS), 392 (ν_a, SAuS), 355 (ν_s, SAuS), 246 (ν_a, BrAuBr), 225 (ν_s, BrAuBr) cm^{-1}. ^{1}H NMR (400 MHz, DMSO-D$_6$, 298 K): δ 7.86 (N***H***, d, $^3J_{NH,H2}$ = 8.2 Hz, 1H), 4.52 (C^1***H***, d, $^3J_{H1,H2}$ = 3.5 Hz, 1H), 4.13 (C$^{2',6'}$***H***$_{eq}$, br dt, $^2J_{H2'/6'eq,H2'/6'ax}$ = 13.5 Hz/$^3J_{H2'/6'eq,H3'/5'}$ = 3.6 Hz, 2H), 3.68-3.29 (C$^{2',6'}$***H***$_{ax}$ + C^2***H*** + C^3***H*** + C^5***H*** + C^6***H***$_2$ + C^{3}O***H*** + C^{4}O***H*** + C^{6}O***H*** overlapped, m, 10H), 3.24 (OC***H***$_3$, s, 3H), 3.12 (C^4***H***, dd, $^3J_{H4,H5}$ = 9.6 Hz/$^3J_{H4,H3}$ = 8.8 Hz, 1H), 2.75 (C$^{4'}$***H***, tt, $^3J_{H4',H3'/5'ax}$ = 10.6 Hz/$^3J_{H4',H3'/5'eq}$ = 3.9 Hz, 1H), 1.95 (C$^{3',5'}$***H***$_{eq}$, td, $^2J_{H3'/5'eq,H3'/5'ax}$ = 12.9 Hz/$^3J_{H3'/5'eq,H2'/6'}$ = 2.6 Hz, 2H), 1.71 (C$^{3',5'}$***H***$_{ax}$, qd, $^2J_{H3'/5'ax,H3'/5'eq}$ = 11.5 Hz/$^3J_{H3'/5'ax,H2'/6'}$ = 4.1 Hz, 2H) ppm. ^{13}C{^{1}H} NMR (100 MHz; DMSO-D$_6$, 298 K): δ 186.9 (N***C***SS), 172.9 (***C***=O), 97.8 (***C***^{1}H), 72.8 (***C***^{5}H), 70.8 (***C***^{3}H), 70.7 (***C***^{4}H), 60.8 (***C***^{6}H$_2$), 54.4 (O***C***H$_3$), 53.7 (***C***^{2}H), 48.4 (***C***$^{2',6'}$H$_2$), 40.3 (***C***$^{4'}$H), 27.7 (***C***$^{3',5'}$H$_2$) ppm. No NMR signals assignable to the β anomer were detected. UV-Vis (DMSO, 50 μM, 298 K): λ_{max} (log ε) 268 (4.25, π*←π intraligand/NCSS), 280 (4.31, π*←π intraligand/NCSS), 315 (4.09, π*←d MLCT), 368$_{sh}$ (3.42, d←d Au(III)) nm.

[Au^{III}Br$_2$(SSC-Inp-GlcN3)] (Au5). FT-IR (CsI disk, 298 K): $\tilde{\nu}_{max}$ 3401 (ν, OH + NH overlapped), 1650 (ν, C=O (amide I)), 1555 (ν, N–CSS), 1538 (δ_{ip}, CNH (amide II)), 1077/1050 (ν, C–OH + C^1–O–CH_3 overlapped), 1011 (ν_a, SCS), 541 (ν_s, SCS), 398 (ν_a, SAuS), 351 (ν_s, SAuS), 254 (ν_a, BrAuBr), 227 (ν_s, BrAuBr) cm^{-1}. ^{1}H NMR (400 MHz, DMSO-D$_6$, 298 K): δ 7.99 (N***H***, t, $^3J_{NH,H6(dia)}$ = 5.7 Hz, 1H), 4.51 (C^1***H***, d, $^3J_{H1,H2}$ = 3.6 Hz, 1H), 4.13 (C$^{2',6'}$***H***$_{eq}$, br dt, $^2J_{H2'/6'eq,H2'/6'ax}$ = 13.4 Hz/$^3J_{H2'/6'eq,H3'/5'}$ = 3.7 Hz, 2H), ~3.6 (C^{2}O***H*** + C^{3}O***H*** + C^{4}O***H*** overlapped, vbr s, 3H), 3.59-3.52 (C^6***H***^{b} + C$^{2',6'}$***H***$_{ax}$ overlapped, m, 3H), 3.37-3.33 (C^3***H*** + C^5***H*** overlapped, m, 2H), 3.24 (OC***H***$_3$, s, 3H), 3.19 (C^2***H***, dd, $^3J_{H2,H3}$ = 9.6 Hz/$^3J_{H2,H1}$ = 3.6 Hz, 1H), 3.01 (C^6***H***^{a}, ddd, $^2J_{H6b,H6a}$ = 13.6 Hz/$^3J_{H6b,NH}$ = 7.4 Hz/$^3J_{H6b,H5}$ = 5.8 Hz, 1H), 2.90 (C^4***H***, br t, $^3J_{H4,H3}$ = 9.2 Hz, 1H), 2.71 (C$^{4'}$***H***, $^3J_{H4',H3'/5'ax}$ = 10.7 Hz/$^3J_{H4',H3'/5'eq}$ = 3.7 Hz, 1H), 1.93 (C$^{3',5'}$***H***$_{eq}$, dt, $^2J_{H3'/5'eq,H3'/5'ax}$ = 13.7 Hz/$^3J_{H3'/5'eq,H2'/6'}$ = 3.8 Hz, 2H), 1.71 (C$^{3',5'}$***H***$_{ax}$, br qd, $^2J_{H3'/5'ax,H3'/5'eq}$ = 11.3 Hz/$^3J_{H3'/5'ax,H2'/6'}$ = 3.5 Hz, 2H) ppm. ^{13}C{^{1}H} NMR (100 MHz; DMSO-D$_6$, 298 K): δ 187.0 (N***C***SS), 172.7 (***C***=O), 99.7 (***C***^{1}H), 73.0 (***C***^{3}H), 72.1 (***C***^{4}H), 71.9 (***C***^{2}H), 70.3 (***C***^{5}H), 54.3 (O***C***H$_3$), 48.4 (***C***$^{2',6'}$H$_2$), 40.5 (***C***$^{4'}$H), 40.1 (***C***^{6}H$_2$), 27.7 (***C***$^{3',5'}$H$_2$) ppm. UV-Vis (DMSO, 50 μM, 298 K): λ_{max} (log ε) 264 (4.11, π*←π intraligand/NCSS), 278 (4.19, π*←π intraligand/NCSS), 313 (3.94, π*←d MLCT), 367$_{sh}$ (3.32, d←d Au(III)) nm.

Synthesis and characterization of the gold(I)-phosphine complexes AuP4-5

The gold(I)-phosphine dithiocarbamato complexes **AuP4** and **AuP5** were synthesized as previously reported in the literature.[38]

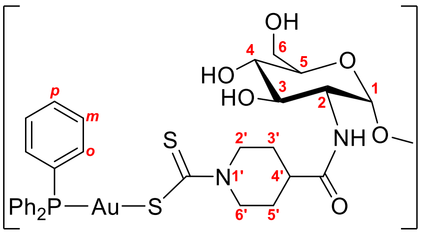

AuP4

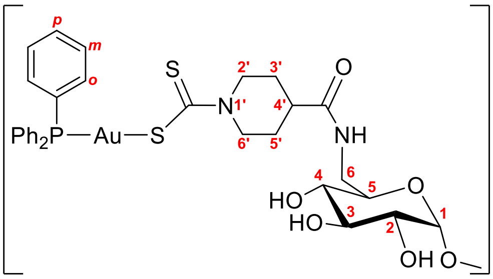

AuP5

[Au^{I}(SSC-Inp-GlcN2)(PPh$_3$)] (AuP4)

Pale yellow solid (85.3 mg, 68% yield). M.p. 178-190°C (dec.). Elemental analysis (%) calcd for $C_{32}H_{38}AuN_2O_6PS_2$ (MM = 838.72 g mol^{-1}): C, 45.83; H, 4.57; N, 3.34; found: C, 46.07; H, 4.86; N, 3.76. FT-IR (CsI disk, 298 K): $\tilde{\nu}_{max}$ 3421 (ν, OH + NH overlapped), 1651 (ν, C=O (amide I)), 1544 (δ_{ip}, CNH (amide II)), 1486 (ν, N–CSS), 1101 ($\nu_{q\ vib}$, P–Ph$_3$), 1056/1043 (ν, C–OH + C^1–O–CH$_3$ overlapped), 999/949 (ν, S=C–S), 710/694 ($\nu_{r\ vib}$, P–Ph$_3$), 539/500 ($\delta_{y\ vib}$, P–Ph$_3$), 510 (ν, C–S), 437/427 ($\nu_{t\ vib}$, P–Ph$_3$), 380 (ν, Au–P), 372 (ν, Au–S), 276/250 ($\delta_{x\ vib}$, P–Ph$_3$) cm^{-1}. ^{1}H NMR (400 MHz, DMSO-D$_6$, 298 K): δ 7.80 (N***H***, d, $^3J_{NH,H2}$ = 8.1 Hz, 1H), 7.62-7.55 (C***H*** Ph, m, 15H), 5.01 (C^{4}O***H***, d, $^3J_{OH4,H4}$ = 5.6 Hz, 1H), 4.90 (C$^{2',6'}$***H***$_{eq}$, br d, $^2J_{H2'/6'eq,H2'/6'ax}$ = 11.6 Hz, 2H), 4.72 (C^{3}O***H***, d, $^3J_{OH3,H3}$ = 6.0 Hz, 1H), 4.55 (C^{6}O***H***, t, $^3J_{OH6,H6(dia)}$ = 6.0 Hz, 1H), 4.53 (C^1***H***, d, $^3J_{H1,H2}$ = 3.4 Hz, 1H), 3.68-3.62 (C^2***H*** + C^6***H***^{b} overlapped, m, 2H), 3.49-3.42 (C^3***H*** + C^6***H***^{a} overlapped, m, 2H), 3.33-3.27 (C^5***H*** + C$^{2',6'}$***H***$_{ax}$ overlapped, m, 3H), 3.24 (OC***H***$_3$, s, 3H), 3.12 (C^4***H***, ddd, $^3J_{H4,H5}$ = 10.3 Hz/$^3J_{H4,H3}$ = 8.9 Hz/$^3J_{H3,OH3}$ = 5.6 Hz, 1H), 2.58 (C$^{4'}$***H***, br t, $^3J_{H4',H3'/5'ax}$ = 10.8 Hz, 1H), 1.79 (C$^{3',5'}$***H***$_{eq}$, br t, $^2J_{H3'/5'eq,H3'/5'ax}$ = 11.1 Hz, 2H), 1.60 (C$^{3',5'}$***H***$_{ax}$, br q, $^2J_{H3'/5'ax,H3'/5'eq}$ = 10.7 Hz, 2H) ppm. ^{13}C{^{1}H} NMR (100 MHz; DMSO-D$_6$, 298 K): δ 202.2 (N***C***SS), 173.9 (***C***=O), 133.7 (*o*-***C***H, d, $^2J_{C,P}$ = 14.1 Hz), 131.9 (*p*-***C***H, br s, $^4J_{C,P}$ = not detectable), 130.1 (***C***P, d, $^1J_{C,P}$ = not detectable due to overlapping), 129.5 (*m*-***C***H, d, $^3J_{C,P}$ = 11.3 Hz), 97.9 (***C***^{1}H), 72.8 (***C***^{5}H), 70.8 (***C***^{3}H), 70.7 (***C***^{4}H), 60.8 (***C***^{6}H$_2$), 54.5 (O***C***H$_3$), 53.8 (***C***^{2}H), 50.8 (***C***$^{2',6'}$H$_2$), 40.4 (***C***$^{4'}$H), 28.5 (***C***$^{3',5'}$H$_2$) ppm. ^{31}P{^{1}H} NMR (162 MHz; DMSO-D$_6$, 298 K): δ 36.4 (Au***P***Ph$_3$) ppm. No NMR signals undoubtedly assignable to the β anomer were detected. UV-Vis (DMSO, 25 μM, 298 K): λ_{max} (log ε) 270 (4.47, π*←π intraligand/NCSS + π*←π & π*←n intraligand/PPh$_3$ overlapped), 294 (4.09, π*←π intraligand/NCSS), 315$_{sh}$ (3.84, p(Au)←p(S) LMCT) nm.[60,61]

[Au^{I}(SSC-Inp-GlcN3)(PPh$_3$)] (AuP5)

Pale yellow solid (95.2 mg, 70% yield). M.p. 162-165°C (dec.). Elemental analysis (%) calcd for $C_{32}H_{38}AuN_2O_6PS_2$ (MM = 838.72 g mol^{-1}): C, 45.83; H, 4.57; N, 3.34; found: C, 46.16; H, 4.56; N, 3.39.

FT-IR (CsI disk, 298 K): $\tilde{\nu}_{max}$ 3429 (ν, OH + NH overlapped), 1654 (ν, C=O (amide I)), 1544 (δ_{ip}, CNH (amide II)), 1483 (ν, N–CSS), 1101 ($\nu_{q\ vib}$, P–Ph$_3$), 1051 (ν, C–OH + C^1–O–CH$_3$ overlapped), 1000/956 (ν, S=C–S), 711/694 ($\nu_{r\ vib}$, P–Ph$_3$), 539/500 ($\delta_{y\ vib}$, P–Ph$_3$), 510 (ν, C–S), 437 ($\nu_{t\ vib}$, P–Ph$_3$), 397 (ν, Au–P), 340 (ν, Au–S), 266/255 ($\delta_{x\ vib}$, P–Ph$_3$) cm^{-1}. ^{1}H NMR (400 MHz, DMSO-D$_6$, 298 K): δ 7.98 (N***H***, t, $^3J_{NH,H6(dia)}$ = 5.7 Hz, 1H), 7.63-7.55 (C***H*** Ph, m, 15H), 4.98 (C^{4}O***H***, d, $^3J_{OH4,H4}$ = 5.5 Hz, 1H), 4.90 (C$^{2',6'}$***H***$_{eq}$, br d, $^2J_{H2'/6'eq,H2'/6'ax}$ = 12.4 Hz, 2H), 4.83 (C^{3}O***H***, d, $^3J_{OH3,H3}$ = 4.8 Hz, 1H), 4.76 (C^{2}O***H***, d, $^3J_{OH2,H2}$ = 6.5 Hz, 1H), 4.51 (C^1***H***, d, $^3J_{H1,H2}$ = 3.6 Hz, 1H), 3.55 (C^6***H***^{b}, ddd, $^2J_{H6b,H6a}$ = 13.5 Hz/$^3J_{H6b,NH}$ = 5.6 Hz/$^3J_{H6b,H5}$ = 1.9 Hz, 1H), 3.38-3.33 (C^3***H*** + C^5***H*** overlapped, m, 2H), 3.24 (OC***H***$_3$, s, 3H), 3.28-3.17 (C^2***H*** + C$^{2',6'}$***H***$_{ax}$ overlapped, m, 3H), 3.01 (C^6***H***^{a}, ddd, $^2J_{H6b,H6a}$ = 13.8 Hz/$^3J_{H6b,NH}$ = 6.0 Hz/$^3J_{H6b,H5}$ = 1.7 Hz, 1H), 2.90 (C^4***H***, ddd, $^3J_{H4,H5}$ = 9.2 Hz/$^3J_{H4,H3}$ = 9.4 Hz/$^3J_{H4,OH4}$ = 5.5 Hz, 1H), 2.57-2.50 (C$^{4'}$***H***, m, 1H), 1.77 (C$^{3',5'}$***H***$_{eq}$, br d, $^2J_{H3'/5'eq,H3'/5'ax}$ = 12.6 Hz, 2H), 1.60 (C$^{3',5'}$***H***$_{ax}$, br q, $^2J_{H3'/5'ax,H3'/5'eq}$ = 11.5 Hz, 2H) ppm. ^{13}C{^{1}H} NMR (100 MHz; DMSO-D$_6$, 298 K): δ 203.7 (N***C***SS), 173.9 (***C***=O), 133.7 (*o*-***C***H, d, $^2J_{C,P}$ = 14.2 Hz), 131.9 (*p*-***C***H, br s, $^4J_{C,P}$ = not detectable), 130.1 (***C***P, d, $^1J_{C,P}$ = not detectable due to overlapping), 129.5 (*m*-***C***H, d, $^3J_{C,P}$ = 11.2 Hz), 99.6 (***C***^{1}H), 73.0 (***C***^{3}H), 72.1 (***C***^{4}H), 72.0 (***C***^{2}H), 70.3 (***C***^{5}H), 54.3 (O***C***H$_3$), 51.1 (***C***$^{2',6'}$H$_2$), 40.5 (***C***$^{4'}$H), 39.9 (***C***^{6}H$_2$), 28.5 (***C***$^{3',5'}$H$_2$) ppm. ^{31}P{^{1}H} NMR (162 MHz; DMSO-D$_6$, 298 K): δ 36.5 (Au***P***Ph$_3$) ppm. UV-Vis (DMSO, 25 μM, 298 K): λ_{max} (log ε) 271 (4.29, π*←π intraligand/NCSS + π*←π & π*←n intraligand/PPh$_3$ overlapped), 295 (3.95, π*←π intraligand/NCSS), 315$_{sh}$ (3.68, p(Au)←p(S) LMCT) nm.

Synthesis, characterization and crystallographic data of the ligand (PPh_4)(SSC-Sar-OEt) (CCDC 2456096)

(PPh_4)(SSC-Sar-OEt)

NaOH (260.7 mg, 6.51 mmol) was dissolved in water (4 mL) and added under stirring to an aqueous solution of ethylsarcosine hydrochloride (996.3 mg, 6.49 mmol). The mixture was cooled down to 0°C, treated dropwise with CS_2 (400 μL, 6.62 mmol), and stirred for 3 h (during which time the pH turned from 9 to 6). The solution was then treated dropwise with an aqueous (10 mL) solution of PPh_4Cl (1.22 g, 3.25 mmol), leading to the sudden precipitation of an off-white solid. The precipitate was centrifuged and the bulk of supernatant discarded. The residue was subsequently washed with cold water (2×5 mL) and then dried under vacuum over P_2O_5, yielding the title compound as a pale yellow solid (1.09 g, 32% yield). Yellow crystals suitable for X-ray crystallography were obtained upon slow evaporation of an aqueous solution of the compound.

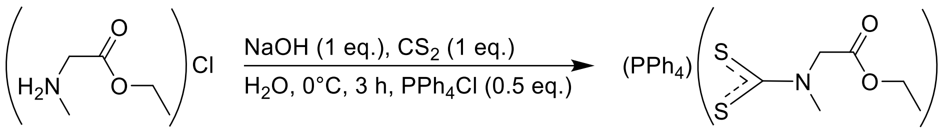

ethylsarcosine hydrochloride **(PPh_4)(SSC-Sar-OEt)**

M.p. 155-160°C (dec.). Elemental analysis (%) calcd for $C_{30}H_{30}NO_2PS_2$ (MM = 531.67 g mol^{-1}): C, 67.77; H, 5.69; N, 2.63; found: C, 68.04; H, 5.55; N, 2.46. FT-IR (CsI disk, 298 K): $\tilde{\nu}_{max}$ 1732 (ν, C=O), 1470 (ν, N–CSS), 1194 (ν, C–OEt), 1107 ($\nu_{q\ vib}$, P–Ph_4), 1030 (ν, O–Et), 995 (ν_a, SCS), 713 ($\nu_{r\ vib}$, P–Ph_4), 688 ($\delta_{v\ vib}$, P–Ph_4), 527 ($\delta_{y\ vib}$, P–Ph_4 + ν, C–S overlapped), 448 ($\nu_{t\ vib}$, P–Ph_4) cm^{-1}. ^{1}H NMR (400 MHz, DMSO-D_6, 298 K): δ 7.91-7.87 (*p*-C***H*** Ph, m, 4H), 7.80-7.75 (*o*-C***H*** Ph, m, 8H), 7.64-7.59 (*m*-C***H*** Ph, m, 8H), 5.12 (NC***H***$_2$, s, 2H), 4.07 (OC***H***$_2$, q, $^3J_{H,H}$ = 7.1 Hz, 2H), 3.54 (NC***H***$_3$, s, 3H), 1.17 (C***H***$_3$, t, $^3J_{H,H}$ = 7.1 Hz, 3H) ppm. ^{13}C{^{1}H} NMR (100 MHz; $CDCl_3$, 298 K): δ 217.2 (N***C***SS), 170.3 (***C***=O), 135.9 (***p-C***H, d, $^4J_{C,P}$ = 2.9 Hz), 134.5 (***m-C***H, d, $^3J_{C,P}$ = 10.4 Hz), 130.9 (***o-C***H, d, $^2J_{C,P}$ = 12.8 Hz), 117.5 (***C***P, d, $^1J_{C,P}$ = 89.3 Hz), 60.3 (O***C***'H_2), 57.5 (N***C***'H_2), 42.8 (N***C***'H_3), 14.3 (***C***'H_3) ppm.

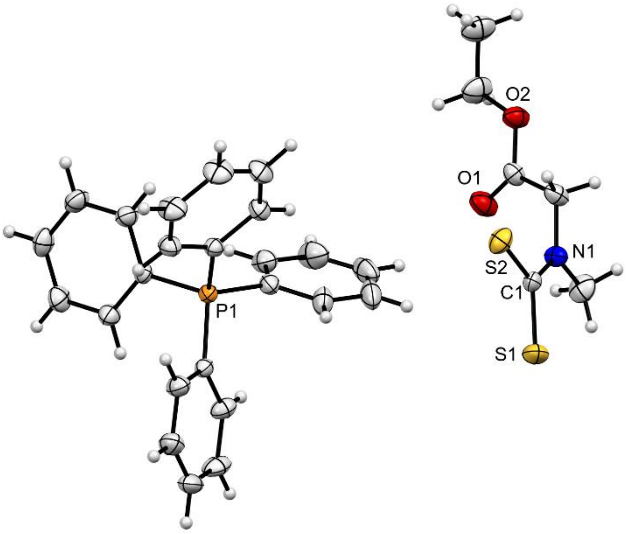

Figure S1. Molecular structure of (PPh_4)(SSC-Sar-OEt). Thermal ellipsoids are depicted at the 30% probability level.

Table S1. Crystal data and structure refinement for (PPh_4)(SSC-Sar-OEt).

Empirical formula	$C_{30}H_{30}NO_2PS_2$	μ/mm^{-1}	0.275
Formula weight	531.64	F(000)	560.0
Temperature/K	297.0(1)	Crystal size/mm^3	0.5 × 0.4 × 0.35
Crystal system	Triclinic	Radiation	MoKα (λ = 0.71073 Å)
Space group	P-1	2Θ range for data collection/°	6.882 to 58.37
a/Å	10.1305(6)	Index ranges	$-13 \leq h \leq 12$, $-14 \leq k \leq 13$, $-17 \leq l \leq 18$
b/Å	11.0650(5)	Reflections collected	11765
c/Å	13.6416(6)	Independent reflections	6352 [R_{int} = 0.0223, R_{sigma} = 0.0433]
α/°	83.631(4)	Data/restraints/parameters	6352/0/327
β/°	71.711(5)	Goodness-of-fit on F^2	1.016
γ/°	74.528(5)	Final R indexes [I>=2σ (I)]	R_1 = 0.0499, wR_2 = 0.1145
Volume/Å^3	1398.67(13)	Final R indexes [all data]	R_1 = 0.0795, wR_2 = 0.1337
Z	2	Largest diff. peak/hole/e Å^{-3}	0.49/-0.35
ρ_{calc}/g cm^{-3}	1.262		

Crystallographic data of [Au^{I}(SSC-Inp-NH_2)(PPh_3)] (AuP2, CCDC 2456093), [Au^{I}(SSC-Sar-OEt)(Et_2BzImy)] (AuC1, CCDC 2456094) and [Au^{I}(SSC-Inp-NH_2)(Et_2BzImy)] (AuC2, CCDC 2456095)

Table S2. Crystal data and structure refinement for **AuP2**, **AuC1** and **AuC2**.

	**AuP2**	**AuC1**	**AuC2**
Empirical formula	$C_{25}H_{26}AuN_2OPS_2$	$C_{17}H_{24}N_3S_2AuO_2$	$C_{18}H_{25}AuN_4OS_2$
Formula weight	662.53	563.48	574.51
Temperature/K	200.01	293(2)	200.00
Crystal system	monoclinic	Monoclinic	monoclinic
Space group	P21	Pn	$P2_1/n$
a/Å	9.8812(3)	4.971(3)	16.3371(5)
b/Å	16.9091(4)	11.096(8)	7.3801(2)
c/Å	15.5657(5)	18.762(10)	17.3336(5)
α/°	90	90	90
β/°	107.2230(10)	90.727(10)	97.1320(10)
γ/°	90	90	90
Volume/$Å^3$	2484.13(13)	1034.8(11)	2073.73(10)
Z	4	2	4
ρ_{calc}/g cm^{-3}	1.772	1.808	1.840
μ/mm^{-1}	6.175	7.325	7.310
F(000)	1296.0	548.0	1120.0
Crystal size/mm^3	0.22 × 0.18 × 0.18	0.32 × 0.14 × 0.13	0.22 × 0.12 × 0.04
Radiation	MoKα (λ = 0.71073 Å)	MoKα (λ = 0.71073 Å)	MoKα (λ = 0.71073 Å)
2Θ range for data collection/°	5.544 to 52.742	3.67 to 53.144	6.398 to 52.064
Index ranges	-12 ≤ h ≤ 12, -21 ≤ k ≤ 21, -19 ≤ l ≤ 19	-6 ≤ h ≤ 6, -13 ≤ k ≤ 13, -23 ≤ l ≤ 23	-20 ≤ h ≤ 20, -9 ≤ k ≤ 9, -21 ≤ l ≤ 20
Reflections collected	76929	12120	25882
Independent reflections	10043 [R_{int} = 0.0375, R_{sigma} = 0.0305]	4289 [R_{int} = 0.0487, R_{sigma}= 0.0643]	4047 [R_{int} = 0.0360, R_{sigma} = 0.0229]
Data/restraints/parameters	10043/1/578	4289/62/256	4047/0/237
Goodness-of-fit on F^2	1.063	0.992	1.068
Final R indexes [I>=2σ (I)]	R_1 = 0.0170, wR_2 = 0.0389	R_1= 0.0359, wR_2= 0.0666	R_1 = 0.0171, wR_2 = 0.0427
Final R indexes [all data]	R_1 = 0.0179, wR_2 = 0.0393	R_1= 0.0474, wR_2= 0.0709	R_1 = 0.0205, wR_2 = 0.0444
Largest diff. peak/hole/e $Å^{-3}$	0.65/-1.11	0.90/-0.46	0.67/-0.59
Flack parameter	0.017(4)	0.020(10)	

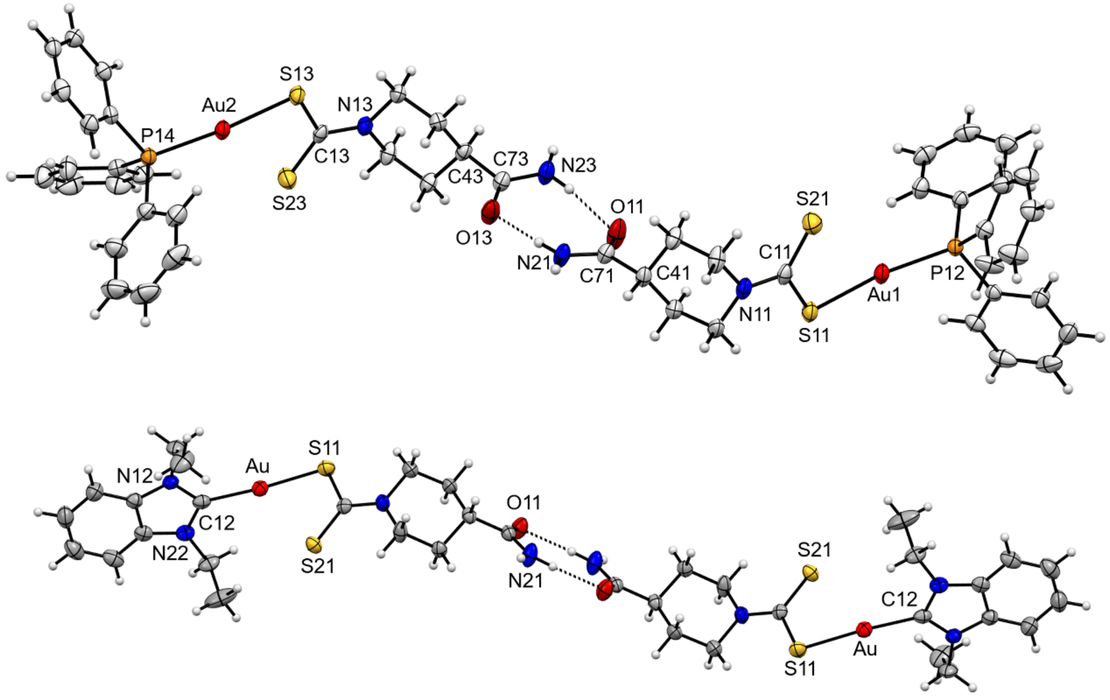

Figure S2. Hydrogen bond dimer comprising the asymmetric unit of **AuP2** (top). Hydrogen bond dimer formed by two symmetry related molecules of **AuC2** (bottom). Thermal ellipsoids are depicted at the 30% probability level. Two molecular entities comprise the asymmetric unit.

Table S3. Selected bond lengths (Å) for **AuP2**, **AuC1** and **AuC2**.

**AuP2**		**AuC1**		**AuC2**	
Au1–S11	2.328(1)	Au–S11	2.288(3)	Au–S11	2.3078(8)
Au1–P12	2.250(1)	Au–C12	1.99(1)	Au–C12	1.995(3)
Au2–S13	2.335(1)	C11–S11	1.74(1)	C11–S11	1.744(3)
Au2–P14	2.254(1)	C11–S12	1.68(1)	C11–S21	1.700(3)
C11–S11	1.751(5)	C11–N11	1.34(1)	C11–N11	1.337(4)
C11–S21	1.687(5)	C12–N22	1.33(1)	C12–N12	1.353(3)
C11–N11	1.340(6)	C12–N12	1.37(1)	C12–N22	1.381(4)
C71–N21	1.318(6)	N11–C21	1.45(2)	C71–O11	1.224(3)
C71–O11	1.231(6)			C71–N21	1.333(4)
C13–S13	1.756(5)				
C13–S23	1.691(5)				
C13–N13	1.333(6)				
C73–N23	1.326(6)				
C73–O13	1.229(6)				
